# Supplementary material for: OpenMM 7: Rapid development of high performance algorithms for molecular dynamics
Source: PLoS Comput Biol. 2017 Jul 26;13(7):e1005659. doi: 10.1371/journal.pcbi.1005659 (PMC5549999; doi:10.1371/journal.pcbi.1005659)
Supplement: S1 Source Code — (ZIP) [file pcbi.1005659.s002.zip › OpenMM-7.0.1-Source/docs/api-python/genindex.html]

Index — OpenMM 7.0.1.dev-5e86c4f documentation


# Index

**\_**
| **A**
| **B**
| **C**
| **D**
| **E**
| **F**
| **G**
| **H**
| **I**
| **L**
| **M**
| **N**
| **O**
| **P**
| **R**
| **S**
| **T**
| **U**
| **V**
| **W**
| **X**

## \_

|  |  |
| --- | --- |
| \_\_add\_\_() (simtk.openmm.vec3.Vec3 method)  \_\_contains\_\_ (simtk.openmm.vec3.Vec3 attribute)  \_\_copy\_\_() (simtk.openmm.openmm.Force method)  (simtk.openmm.openmm.Integrator method)  (simtk.openmm.openmm.System method)  \_\_delattr\_\_ (simtk.openmm.amd.AMDForceGroupIntegrator attribute)  (simtk.openmm.amd.AMDIntegrator attribute)  (simtk.openmm.amd.DualAMDIntegrator attribute)  (simtk.openmm.app.amberinpcrdfile.AmberInpcrdFile attribute)  (simtk.openmm.app.amberprmtopfile.AmberPrmtopFile attribute)  (simtk.openmm.app.charmmcrdfiles.CharmmCrdFile attribute)  (simtk.openmm.app.charmmcrdfiles.CharmmRstFile attribute)  (simtk.openmm.app.charmmparameterset.CharmmParameterSet attribute)  (simtk.openmm.app.charmmpsffile.CharmmPsfFile attribute)  (simtk.openmm.app.checkpointreporter.CheckpointReporter attribute)  (simtk.openmm.app.dcdfile.DCDFile attribute)  (simtk.openmm.app.dcdreporter.DCDReporter attribute)  (simtk.openmm.app.desmonddmsfile.DesmondDMSFile attribute)  (simtk.openmm.app.element.Element attribute)  (simtk.openmm.app.forcefield.ForceField attribute)  (simtk.openmm.app.gromacsgrofile.GromacsGroFile attribute)  (simtk.openmm.app.gromacstopfile.GromacsTopFile attribute)  (simtk.openmm.app.internal.charmm.exceptions.CharmmPSFWarning attribute)  (simtk.openmm.app.modeller.Modeller attribute)  (simtk.openmm.app.pdbfile.PDBFile attribute)  (simtk.openmm.app.pdbreporter.PDBReporter attribute)  (simtk.openmm.app.pdbreporter.PDBxReporter attribute)  (simtk.openmm.app.pdbxfile.PDBxFile attribute)  (simtk.openmm.app.simulation.Simulation attribute)  (simtk.openmm.app.statedatareporter.StateDataReporter attribute)  (simtk.openmm.app.topology.Atom attribute)  (simtk.openmm.app.topology.Chain attribute)  (simtk.openmm.app.topology.Residue attribute)  (simtk.openmm.app.topology.Topology attribute)  (simtk.openmm.mtsintegrator.MTSIntegrator attribute)  (simtk.openmm.openmm.AmoebaAngleForce attribute)  (simtk.openmm.openmm.AmoebaBondForce attribute)  (simtk.openmm.openmm.AmoebaGeneralizedKirkwoodForce attribute)  (simtk.openmm.openmm.AmoebaInPlaneAngleForce attribute)  (simtk.openmm.openmm.AmoebaMultipoleForce attribute)  (simtk.openmm.openmm.AmoebaOutOfPlaneBendForce attribute)  (simtk.openmm.openmm.AmoebaPiTorsionForce attribute)  (simtk.openmm.openmm.AmoebaStretchBendForce attribute)  (simtk.openmm.openmm.AmoebaTorsionTorsionForce attribute)  (simtk.openmm.openmm.AmoebaVdwForce attribute)  (simtk.openmm.openmm.AmoebaWcaDispersionForce attribute)  (simtk.openmm.openmm.AndersenThermostat attribute)  (simtk.openmm.openmm.BrownianIntegrator attribute)  (simtk.openmm.openmm.CMAPTorsionForce attribute)  (simtk.openmm.openmm.CMMotionRemover attribute)  (simtk.openmm.openmm.CompoundIntegrator attribute)  (simtk.openmm.openmm.Context attribute)  (simtk.openmm.openmm.Continuous1DFunction attribute)  (simtk.openmm.openmm.Continuous2DFunction attribute)  (simtk.openmm.openmm.Continuous3DFunction attribute)  (simtk.openmm.openmm.CustomAngleForce attribute)  (simtk.openmm.openmm.CustomBondForce attribute)  (simtk.openmm.openmm.CustomCentroidBondForce attribute)  (simtk.openmm.openmm.CustomCompoundBondForce attribute)  (simtk.openmm.openmm.CustomExternalForce attribute)  (simtk.openmm.openmm.CustomGBForce attribute)  (simtk.openmm.openmm.CustomHbondForce attribute)  (simtk.openmm.openmm.CustomIntegrator attribute)  (simtk.openmm.openmm.CustomManyParticleForce attribute)  (simtk.openmm.openmm.CustomNonbondedForce attribute)  (simtk.openmm.openmm.CustomTorsionForce attribute)  (simtk.openmm.openmm.Discrete1DFunction attribute)  (simtk.openmm.openmm.Discrete2DFunction attribute)  (simtk.openmm.openmm.Discrete3DFunction attribute)  (simtk.openmm.openmm.DrudeForce attribute)  (simtk.openmm.openmm.DrudeLangevinIntegrator attribute)  (simtk.openmm.openmm.DrudeSCFIntegrator attribute)  (simtk.openmm.openmm.Force attribute)  (simtk.openmm.openmm.GBSAOBCForce attribute)  (simtk.openmm.openmm.HarmonicAngleForce attribute)  (simtk.openmm.openmm.HarmonicBondForce attribute)  (simtk.openmm.openmm.Integrator attribute)  (simtk.openmm.openmm.LangevinIntegrator attribute)  (simtk.openmm.openmm.LocalCoordinatesSite attribute)  (simtk.openmm.openmm.LocalEnergyMinimizer attribute)  (simtk.openmm.openmm.MonteCarloAnisotropicBarostat attribute)  (simtk.openmm.openmm.MonteCarloBarostat attribute)  (simtk.openmm.openmm.MonteCarloMembraneBarostat attribute)  (simtk.openmm.openmm.NonbondedForce attribute)  (simtk.openmm.openmm.OutOfPlaneSite attribute)  (simtk.openmm.openmm.PeriodicTorsionForce attribute)  (simtk.openmm.openmm.Platform attribute)  (simtk.openmm.openmm.RBTorsionForce attribute)  (simtk.openmm.openmm.RPMDIntegrator attribute)  (simtk.openmm.openmm.RPMDMonteCarloBarostat attribute)  (simtk.openmm.openmm.SerializationNode attribute)  (simtk.openmm.openmm.SerializationProxy attribute)  (simtk.openmm.openmm.State attribute)  (simtk.openmm.openmm.System attribute)  (simtk.openmm.openmm.TabulatedFunction attribute)  (simtk.openmm.openmm.ThreeParticleAverageSite attribute)  (simtk.openmm.openmm.TwoParticleAverageSite attribute)  (simtk.openmm.openmm.VariableLangevinIntegrator attribute)  (simtk.openmm.openmm.VariableVerletIntegrator attribute)  (simtk.openmm.openmm.VerletIntegrator attribute)  (simtk.openmm.openmm.VirtualSite attribute)  (simtk.openmm.openmm.XmlSerializer attribute)  (simtk.openmm.vec3.Vec3 attribute)  \_\_div\_\_() (simtk.openmm.vec3.Vec3 method)  \_\_eq\_\_ (simtk.openmm.vec3.Vec3 attribute)  \_\_format\_\_() (simtk.openmm.amd.AMDForceGroupIntegrator method)  (simtk.openmm.amd.AMDIntegrator method)  (simtk.openmm.amd.DualAMDIntegrator method)  (simtk.openmm.app.amberinpcrdfile.AmberInpcrdFile method)  (simtk.openmm.app.amberprmtopfile.AmberPrmtopFile method)  (simtk.openmm.app.charmmcrdfiles.CharmmCrdFile method)  (simtk.openmm.app.charmmcrdfiles.CharmmRstFile method)  (simtk.openmm.app.charmmparameterset.CharmmParameterSet method)  (simtk.openmm.app.charmmpsffile.CharmmPsfFile method)  (simtk.openmm.app.checkpointreporter.CheckpointReporter method)  (simtk.openmm.app.dcdfile.DCDFile method)  (simtk.openmm.app.dcdreporter.DCDReporter method)  (simtk.openmm.app.desmonddmsfile.DesmondDMSFile method)  (simtk.openmm.app.element.Element method)  (simtk.openmm.app.forcefield.ForceField method)  (simtk.openmm.app.gromacsgrofile.GromacsGroFile method)  (simtk.openmm.app.gromacstopfile.GromacsTopFile method)  (simtk.openmm.app.internal.charmm.exceptions.CharmmPSFWarning method)  (simtk.openmm.app.modeller.Modeller method)  (simtk.openmm.app.pdbfile.PDBFile method)  (simtk.openmm.app.pdbreporter.PDBReporter method)  (simtk.openmm.app.pdbreporter.PDBxReporter method)  (simtk.openmm.app.pdbxfile.PDBxFile method)  (simtk.openmm.app.simulation.Simulation method)  (simtk.openmm.app.statedatareporter.StateDataReporter method)  (simtk.openmm.app.topology.Atom method)  (simtk.openmm.app.topology.Chain method)  (simtk.openmm.app.topology.Residue method)  (simtk.openmm.app.topology.Topology method)  (simtk.openmm.mtsintegrator.MTSIntegrator method)  (simtk.openmm.openmm.AmoebaAngleForce method)  (simtk.openmm.openmm.AmoebaBondForce method)  (simtk.openmm.openmm.AmoebaGeneralizedKirkwoodForce method)  (simtk.openmm.openmm.AmoebaInPlaneAngleForce method)  (simtk.openmm.openmm.AmoebaMultipoleForce method)  (simtk.openmm.openmm.AmoebaOutOfPlaneBendForce method)  (simtk.openmm.openmm.AmoebaPiTorsionForce method)  (simtk.openmm.openmm.AmoebaStretchBendForce method)  (simtk.openmm.openmm.AmoebaTorsionTorsionForce method)  (simtk.openmm.openmm.AmoebaVdwForce method)  (simtk.openmm.openmm.AmoebaWcaDispersionForce method)  (simtk.openmm.openmm.AndersenThermostat method)  (simtk.openmm.openmm.BrownianIntegrator method)  (simtk.openmm.openmm.CMAPTorsionForce method)  (simtk.openmm.openmm.CMMotionRemover method)  (simtk.openmm.openmm.CompoundIntegrator method)  (simtk.openmm.openmm.Context method)  (simtk.openmm.openmm.Continuous1DFunction method)  (simtk.openmm.openmm.Continuous2DFunction method)  (simtk.openmm.openmm.Continuous3DFunction method)  (simtk.openmm.openmm.CustomAngleForce method)  (simtk.openmm.openmm.CustomBondForce method)  (simtk.openmm.openmm.CustomCentroidBondForce method)  (simtk.openmm.openmm.CustomCompoundBondForce method)  (simtk.openmm.openmm.CustomExternalForce method)  (simtk.openmm.openmm.CustomGBForce method)  (simtk.openmm.openmm.CustomHbondForce method)  (simtk.openmm.openmm.CustomIntegrator method)  (simtk.openmm.openmm.CustomManyParticleForce method)  (simtk.openmm.openmm.CustomNonbondedForce method)  (simtk.openmm.openmm.CustomTorsionForce method)  (simtk.openmm.openmm.Discrete1DFunction method)  (simtk.openmm.openmm.Discrete2DFunction method)  (simtk.openmm.openmm.Discrete3DFunction method)  (simtk.openmm.openmm.DrudeForce method)  (simtk.openmm.openmm.DrudeLangevinIntegrator method)  (simtk.openmm.openmm.DrudeSCFIntegrator method)  (simtk.openmm.openmm.Force method)  (simtk.openmm.openmm.GBSAOBCForce method)  (simtk.openmm.openmm.HarmonicAngleForce method)  (simtk.openmm.openmm.HarmonicBondForce method)  (simtk.openmm.openmm.Integrator method)  (simtk.openmm.openmm.LangevinIntegrator method)  (simtk.openmm.openmm.LocalCoordinatesSite method)  (simtk.openmm.openmm.LocalEnergyMinimizer method)  (simtk.openmm.openmm.MonteCarloAnisotropicBarostat method)  (simtk.openmm.openmm.MonteCarloBarostat method)  (simtk.openmm.openmm.MonteCarloMembraneBarostat method)  (simtk.openmm.openmm.NonbondedForce method)  (simtk.openmm.openmm.OutOfPlaneSite method)  (simtk.openmm.openmm.PeriodicTorsionForce method)  (simtk.openmm.openmm.Platform method)  (simtk.openmm.openmm.RBTorsionForce method)  (simtk.openmm.openmm.RPMDIntegrator method)  (simtk.openmm.openmm.RPMDMonteCarloBarostat method)  (simtk.openmm.openmm.SerializationNode method)  (simtk.openmm.openmm.SerializationProxy method)  (simtk.openmm.openmm.State method)  (simtk.openmm.openmm.System method)  (simtk.openmm.openmm.TabulatedFunction method)  (simtk.openmm.openmm.ThreeParticleAverageSite method)  (simtk.openmm.openmm.TwoParticleAverageSite method)  (simtk.openmm.openmm.VariableLangevinIntegrator method)  (simtk.openmm.openmm.VariableVerletIntegrator method)  (simtk.openmm.openmm.VerletIntegrator method)  (simtk.openmm.openmm.VirtualSite method)  (simtk.openmm.openmm.XmlSerializer method)  (simtk.openmm.vec3.Vec3 method)  \_\_ge\_\_ (simtk.openmm.vec3.Vec3 attribute)  \_\_getattribute\_\_ (simtk.openmm.amd.AMDForceGroupIntegrator attribute)  (simtk.openmm.amd.AMDIntegrator attribute)  (simtk.openmm.amd.DualAMDIntegrator attribute)  (simtk.openmm.app.amberinpcrdfile.AmberInpcrdFile attribute)  (simtk.openmm.app.amberprmtopfile.AmberPrmtopFile attribute)  (simtk.openmm.app.charmmcrdfiles.CharmmCrdFile attribute)  (simtk.openmm.app.charmmcrdfiles.CharmmRstFile attribute)  (simtk.openmm.app.charmmparameterset.CharmmParameterSet attribute)  (simtk.openmm.app.charmmpsffile.CharmmPsfFile attribute)  (simtk.openmm.app.checkpointreporter.CheckpointReporter attribute)  (simtk.openmm.app.dcdfile.DCDFile attribute)  (simtk.openmm.app.dcdreporter.DCDReporter attribute)  (simtk.openmm.app.desmonddmsfile.DesmondDMSFile attribute)  (simtk.openmm.app.element.Element attribute)  (simtk.openmm.app.forcefield.ForceField attribute)  (simtk.openmm.app.gromacsgrofile.GromacsGroFile attribute)  (simtk.openmm.app.gromacstopfile.GromacsTopFile attribute)  (simtk.openmm.app.internal.charmm.exceptions.CharmmPSFWarning attribute)  (simtk.openmm.app.modeller.Modeller attribute)  (simtk.openmm.app.pdbfile.PDBFile attribute)  (simtk.openmm.app.pdbreporter.PDBReporter attribute)  (simtk.openmm.app.pdbreporter.PDBxReporter attribute)  (simtk.openmm.app.pdbxfile.PDBxFile attribute)  (simtk.openmm.app.simulation.Simulation attribute)  (simtk.openmm.app.statedatareporter.StateDataReporter attribute)  (simtk.openmm.app.topology.Atom attribute)  (simtk.openmm.app.topology.Chain attribute)  (simtk.openmm.app.topology.Residue attribute)  (simtk.openmm.app.topology.Topology attribute)  (simtk.openmm.mtsintegrator.MTSIntegrator attribute)  (simtk.openmm.openmm.AmoebaAngleForce attribute)  (simtk.openmm.openmm.AmoebaBondForce attribute)  (simtk.openmm.openmm.AmoebaGeneralizedKirkwoodForce attribute)  (simtk.openmm.openmm.AmoebaInPlaneAngleForce attribute)  (simtk.openmm.openmm.AmoebaMultipoleForce attribute)  (simtk.openmm.openmm.AmoebaOutOfPlaneBendForce attribute)  (simtk.openmm.openmm.AmoebaPiTorsionForce attribute)  (simtk.openmm.openmm.AmoebaStretchBendForce attribute)  (simtk.openmm.openmm.AmoebaTorsionTorsionForce attribute)  (simtk.openmm.openmm.AmoebaVdwForce attribute)  (simtk.openmm.openmm.AmoebaWcaDispersionForce attribute)  (simtk.openmm.openmm.AndersenThermostat attribute)  (simtk.openmm.openmm.BrownianIntegrator attribute)  (simtk.openmm.openmm.CMAPTorsionForce attribute)  (simtk.openmm.openmm.CMMotionRemover attribute)  (simtk.openmm.openmm.CompoundIntegrator attribute)  (simtk.openmm.openmm.Context attribute)  (simtk.openmm.openmm.Continuous1DFunction attribute)  (simtk.openmm.openmm.Continuous2DFunction attribute)  (simtk.openmm.openmm.Continuous3DFunction attribute)  (simtk.openmm.openmm.CustomAngleForce attribute)  (simtk.openmm.openmm.CustomBondForce attribute)  (simtk.openmm.openmm.CustomCentroidBondForce attribute)  (simtk.openmm.openmm.CustomCompoundBondForce attribute)  (simtk.openmm.openmm.CustomExternalForce attribute)  (simtk.openmm.openmm.CustomGBForce attribute)  (simtk.openmm.openmm.CustomHbondForce attribute)  (simtk.openmm.openmm.CustomIntegrator attribute)  (simtk.openmm.openmm.CustomManyParticleForce attribute)  (simtk.openmm.openmm.CustomNonbondedForce attribute)  (simtk.openmm.openmm.CustomTorsionForce attribute)  (simtk.openmm.openmm.Discrete1DFunction attribute)  (simtk.openmm.openmm.Discrete2DFunction attribute)  (simtk.openmm.openmm.Discrete3DFunction attribute)  (simtk.openmm.openmm.DrudeForce attribute)  (simtk.openmm.openmm.DrudeLangevinIntegrator attribute)  (simtk.openmm.openmm.DrudeSCFIntegrator attribute)  (simtk.openmm.openmm.Force attribute)  (simtk.openmm.openmm.GBSAOBCForce attribute)  (simtk.openmm.openmm.HarmonicAngleForce attribute)  (simtk.openmm.openmm.HarmonicBondForce attribute)  (simtk.openmm.openmm.Integrator attribute)  (simtk.openmm.openmm.LangevinIntegrator attribute)  (simtk.openmm.openmm.LocalCoordinatesSite attribute)  (simtk.openmm.openmm.LocalEnergyMinimizer attribute)  (simtk.openmm.openmm.MonteCarloAnisotropicBarostat attribute)  (simtk.openmm.openmm.MonteCarloBarostat attribute)  (simtk.openmm.openmm.MonteCarloMembraneBarostat attribute)  (simtk.openmm.openmm.NonbondedForce attribute)  (simtk.openmm.openmm.OutOfPlaneSite attribute)  (simtk.openmm.openmm.PeriodicTorsionForce attribute)  (simtk.openmm.openmm.Platform attribute)  (simtk.openmm.openmm.RBTorsionForce attribute)  (simtk.openmm.openmm.RPMDIntegrator attribute)  (simtk.openmm.openmm.RPMDMonteCarloBarostat attribute)  (simtk.openmm.openmm.SerializationNode attribute)  (simtk.openmm.openmm.SerializationProxy attribute)  (simtk.openmm.openmm.State attribute)  (simtk.openmm.openmm.System attribute)  (simtk.openmm.openmm.TabulatedFunction attribute)  (simtk.openmm.openmm.ThreeParticleAverageSite attribute)  (simtk.openmm.openmm.TwoParticleAverageSite attribute)  (simtk.openmm.openmm.VariableLangevinIntegrator attribute)  (simtk.openmm.openmm.VariableVerletIntegrator attribute)  (simtk.openmm.openmm.VerletIntegrator attribute)  (simtk.openmm.openmm.VirtualSite attribute)  (simtk.openmm.openmm.XmlSerializer attribute)  (simtk.openmm.vec3.Vec3 attribute)  \_\_getitem\_\_ (simtk.openmm.app.internal.charmm.exceptions.CharmmPSFWarning attribute)  (simtk.openmm.vec3.Vec3 attribute)  \_\_getnewargs\_\_() (simtk.openmm.vec3.Vec3 method)  \_\_getslice\_\_ (simtk.openmm.app.internal.charmm.exceptions.CharmmPSFWarning attribute)  (simtk.openmm.vec3.Vec3 attribute)  \_\_gt\_\_ (simtk.openmm.vec3.Vec3 attribute)  \_\_hash\_\_ (simtk.openmm.amd.AMDForceGroupIntegrator attribute)  (simtk.openmm.amd.AMDIntegrator attribute)  (simtk.openmm.amd.DualAMDIntegrator attribute)  (simtk.openmm.app.amberinpcrdfile.AmberInpcrdFile attribute)  (simtk.openmm.app.amberprmtopfile.AmberPrmtopFile attribute)  (simtk.openmm.app.charmmcrdfiles.CharmmCrdFile attribute)  (simtk.openmm.app.charmmcrdfiles.CharmmRstFile attribute)  (simtk.openmm.app.charmmparameterset.CharmmParameterSet attribute)  (simtk.openmm.app.charmmpsffile.CharmmPsfFile attribute)  (simtk.openmm.app.checkpointreporter.CheckpointReporter attribute)  (simtk.openmm.app.dcdfile.DCDFile attribute)  (simtk.openmm.app.dcdreporter.DCDReporter attribute)  (simtk.openmm.app.desmonddmsfile.DesmondDMSFile attribute)  (simtk.openmm.app.element.Element attribute)  (simtk.openmm.app.forcefield.ForceField attribute)  (simtk.openmm.app.gromacsgrofile.GromacsGroFile attribute)  (simtk.openmm.app.gromacstopfile.GromacsTopFile attribute)  (simtk.openmm.app.internal.charmm.exceptions.CharmmPSFWarning attribute)  (simtk.openmm.app.modeller.Modeller attribute)  (simtk.openmm.app.pdbfile.PDBFile attribute)  (simtk.openmm.app.pdbreporter.PDBReporter attribute)  (simtk.openmm.app.pdbreporter.PDBxReporter attribute)  (simtk.openmm.app.pdbxfile.PDBxFile attribute)  (simtk.openmm.app.simulation.Simulation attribute)  (simtk.openmm.app.statedatareporter.StateDataReporter attribute)  (simtk.openmm.app.topology.Atom attribute)  (simtk.openmm.app.topology.Chain attribute)  (simtk.openmm.app.topology.Residue attribute)  (simtk.openmm.app.topology.Topology attribute)  (simtk.openmm.mtsintegrator.MTSIntegrator attribute)  (simtk.openmm.openmm.AmoebaAngleForce attribute)  (simtk.openmm.openmm.AmoebaBondForce attribute)  (simtk.openmm.openmm.AmoebaGeneralizedKirkwoodForce attribute)  (simtk.openmm.openmm.AmoebaInPlaneAngleForce attribute)  (simtk.openmm.openmm.AmoebaMultipoleForce attribute)  (simtk.openmm.openmm.AmoebaOutOfPlaneBendForce attribute)  (simtk.openmm.openmm.AmoebaPiTorsionForce attribute)  (simtk.openmm.openmm.AmoebaStretchBendForce attribute)  (simtk.openmm.openmm.AmoebaTorsionTorsionForce attribute)  (simtk.openmm.openmm.AmoebaVdwForce attribute)  (simtk.openmm.openmm.AmoebaWcaDispersionForce attribute)  (simtk.openmm.openmm.AndersenThermostat attribute)  (simtk.openmm.openmm.BrownianIntegrator attribute)  (simtk.openmm.openmm.CMAPTorsionForce attribute)  (simtk.openmm.openmm.CMMotionRemover attribute)  (simtk.openmm.openmm.CompoundIntegrator attribute)  (simtk.openmm.openmm.Context attribute)  (simtk.openmm.openmm.Continuous1DFunction attribute)  (simtk.openmm.openmm.Continuous2DFunction attribute)  (simtk.openmm.openmm.Continuous3DFunction attribute)  (simtk.openmm.openmm.CustomAngleForce attribute)  (simtk.openmm.openmm.CustomBondForce attribute)  (simtk.openmm.openmm.CustomCentroidBondForce attribute)  (simtk.openmm.openmm.CustomCompoundBondForce attribute)  (simtk.openmm.openmm.CustomExternalForce attribute)  (simtk.openmm.openmm.CustomGBForce attribute)  (simtk.openmm.openmm.CustomHbondForce attribute)  (simtk.openmm.openmm.CustomIntegrator attribute)  (simtk.openmm.openmm.CustomManyParticleForce attribute)  (simtk.openmm.openmm.CustomNonbondedForce attribute)  (simtk.openmm.openmm.CustomTorsionForce attribute)  (simtk.openmm.openmm.Discrete1DFunction attribute)  (simtk.openmm.openmm.Discrete2DFunction attribute)  (simtk.openmm.openmm.Discrete3DFunction attribute)  (simtk.openmm.openmm.DrudeForce attribute)  (simtk.openmm.openmm.DrudeLangevinIntegrator attribute)  (simtk.openmm.openmm.DrudeSCFIntegrator attribute)  (simtk.openmm.openmm.Force attribute)  (simtk.openmm.openmm.GBSAOBCForce attribute)  (simtk.openmm.openmm.HarmonicAngleForce attribute)  (simtk.openmm.openmm.HarmonicBondForce attribute)  (simtk.openmm.openmm.Integrator attribute)  (simtk.openmm.openmm.LangevinIntegrator attribute)  (simtk.openmm.openmm.LocalCoordinatesSite attribute)  (simtk.openmm.openmm.LocalEnergyMinimizer attribute)  (simtk.openmm.openmm.MonteCarloAnisotropicBarostat attribute)  (simtk.openmm.openmm.MonteCarloBarostat attribute)  (simtk.openmm.openmm.MonteCarloMembraneBarostat attribute)  (simtk.openmm.openmm.NonbondedForce attribute)  (simtk.openmm.openmm.OutOfPlaneSite attribute)  (simtk.openmm.openmm.PeriodicTorsionForce attribute)  (simtk.openmm.openmm.Platform attribute)  (simtk.openmm.openmm.RBTorsionForce attribute)  (simtk.openmm.openmm.RPMDIntegrator attribute)  (simtk.openmm.openmm.RPMDMonteCarloBarostat attribute)  (simtk.openmm.openmm.SerializationNode attribute)  (simtk.openmm.openmm.SerializationProxy attribute)  (simtk.openmm.openmm.State attribute)  (simtk.openmm.openmm.System attribute)  (simtk.openmm.openmm.TabulatedFunction attribute)  (simtk.openmm.openmm.ThreeParticleAverageSite attribute)  (simtk.openmm.openmm.TwoParticleAverageSite attribute)  (simtk.openmm.openmm.VariableLangevinIntegrator attribute)  (simtk.openmm.openmm.VariableVerletIntegrator attribute)  (simtk.openmm.openmm.VerletIntegrator attribute)  (simtk.openmm.openmm.VirtualSite attribute)  (simtk.openmm.openmm.XmlSerializer attribute)  (simtk.openmm.vec3.Vec3 attribute)  \_\_init\_\_() (simtk.openmm.amd.AMDForceGroupIntegrator method)  (simtk.openmm.amd.AMDIntegrator method)  (simtk.openmm.amd.DualAMDIntegrator method)  (simtk.openmm.app.amberinpcrdfile.AmberInpcrdFile method)  (simtk.openmm.app.amberprmtopfile.AmberPrmtopFile method)  (simtk.openmm.app.charmmcrdfiles.CharmmCrdFile method)  (simtk.openmm.app.charmmcrdfiles.CharmmRstFile method)  (simtk.openmm.app.charmmparameterset.CharmmParameterSet method)  (simtk.openmm.app.charmmpsffile.CharmmPsfFile method)  (simtk.openmm.app.checkpointreporter.CheckpointReporter method)  (simtk.openmm.app.dcdfile.DCDFile method)  (simtk.openmm.app.dcdreporter.DCDReporter method)  (simtk.openmm.app.desmonddmsfile.DesmondDMSFile method)  (simtk.openmm.app.element.Element method)  (simtk.openmm.app.forcefield.ForceField method)  (simtk.openmm.app.gromacsgrofile.GromacsGroFile method)  (simtk.openmm.app.gromacstopfile.GromacsTopFile method)  (simtk.openmm.app.internal.charmm.exceptions.CharmmPSFWarning method)  (simtk.openmm.app.modeller.Modeller method)  (simtk.openmm.app.pdbfile.PDBFile method)  (simtk.openmm.app.pdbreporter.PDBReporter method)  (simtk.openmm.app.pdbreporter.PDBxReporter method)  (simtk.openmm.app.pdbxfile.PDBxFile method)  (simtk.openmm.app.simulation.Simulation method)  (simtk.openmm.app.statedatareporter.StateDataReporter method)  (simtk.openmm.app.topology.Atom method)  (simtk.openmm.app.topology.Chain method)  (simtk.openmm.app.topology.Residue method)  (simtk.openmm.app.topology.Topology method)  (simtk.openmm.mtsintegrator.MTSIntegrator method)  (simtk.openmm.openmm.AmoebaAngleForce method)  (simtk.openmm.openmm.AmoebaBondForce method)  (simtk.openmm.openmm.AmoebaGeneralizedKirkwoodForce method)  (simtk.openmm.openmm.AmoebaInPlaneAngleForce method)  (simtk.openmm.openmm.AmoebaMultipoleForce method)  (simtk.openmm.openmm.AmoebaOutOfPlaneBendForce method)  (simtk.openmm.openmm.AmoebaPiTorsionForce method)  (simtk.openmm.openmm.AmoebaStretchBendForce method)  (simtk.openmm.openmm.AmoebaTorsionTorsionForce method)  (simtk.openmm.openmm.AmoebaVdwForce method)  (simtk.openmm.openmm.AmoebaWcaDispersionForce method)  (simtk.openmm.openmm.AndersenThermostat method)  (simtk.openmm.openmm.BrownianIntegrator method)  (simtk.openmm.openmm.CMAPTorsionForce method)  (simtk.openmm.openmm.CMMotionRemover method)  (simtk.openmm.openmm.CompoundIntegrator method)  (simtk.openmm.openmm.Context method)  (simtk.openmm.openmm.Continuous1DFunction method)  (simtk.openmm.openmm.Continuous2DFunction method)  (simtk.openmm.openmm.Continuous3DFunction method)  (simtk.openmm.openmm.CustomAngleForce method)  (simtk.openmm.openmm.CustomBondForce method)  (simtk.openmm.openmm.CustomCentroidBondForce method)  (simtk.openmm.openmm.CustomCompoundBondForce method)  (simtk.openmm.openmm.CustomExternalForce method)  (simtk.openmm.openmm.CustomGBForce method)  (simtk.openmm.openmm.CustomHbondForce method)  (simtk.openmm.openmm.CustomIntegrator method)  (simtk.openmm.openmm.CustomManyParticleForce method)  (simtk.openmm.openmm.CustomNonbondedForce method)  (simtk.openmm.openmm.CustomTorsionForce method)  (simtk.openmm.openmm.Discrete1DFunction method)  (simtk.openmm.openmm.Discrete2DFunction method)  (simtk.openmm.openmm.Discrete3DFunction method)  (simtk.openmm.openmm.DrudeForce method)  (simtk.openmm.openmm.DrudeLangevinIntegrator method)  (simtk.openmm.openmm.DrudeSCFIntegrator method)  (simtk.openmm.openmm.Force method)  (simtk.openmm.openmm.GBSAOBCForce method)  (simtk.openmm.openmm.HarmonicAngleForce method)  (simtk.openmm.openmm.HarmonicBondForce method)  (simtk.openmm.openmm.Integrator method)  (simtk.openmm.openmm.LangevinIntegrator method)  (simtk.openmm.openmm.LocalCoordinatesSite method)  (simtk.openmm.openmm.LocalEnergyMinimizer method)  (simtk.openmm.openmm.MonteCarloAnisotropicBarostat method)  (simtk.openmm.openmm.MonteCarloBarostat method)  (simtk.openmm.openmm.MonteCarloMembraneBarostat method)  (simtk.openmm.openmm.NonbondedForce method)  (simtk.openmm.openmm.OutOfPlaneSite method)  (simtk.openmm.openmm.PeriodicTorsionForce method)  (simtk.openmm.openmm.Platform method)  (simtk.openmm.openmm.RBTorsionForce method)  (simtk.openmm.openmm.RPMDIntegrator method)  (simtk.openmm.openmm.RPMDMonteCarloBarostat method)  (simtk.openmm.openmm.SerializationNode method)  (simtk.openmm.openmm.SerializationProxy method)  (simtk.openmm.openmm.State method)  (simtk.openmm.openmm.System method)  (simtk.openmm.openmm.TabulatedFunction method)  (simtk.openmm.openmm.ThreeParticleAverageSite method)  (simtk.openmm.openmm.TwoParticleAverageSite method)  (simtk.openmm.openmm.VariableLangevinIntegrator method)  (simtk.openmm.openmm.VariableVerletIntegrator method)  (simtk.openmm.openmm.VerletIntegrator method)  (simtk.openmm.openmm.VirtualSite method)  (simtk.openmm.openmm.XmlSerializer method)  (simtk.openmm.vec3.Vec3 method)  \_\_iter\_\_ (simtk.openmm.vec3.Vec3 attribute) | \_\_le\_\_ (simtk.openmm.vec3.Vec3 attribute)  \_\_len\_\_ (simtk.openmm.vec3.Vec3 attribute)  \_\_lt\_\_ (simtk.openmm.vec3.Vec3 attribute)  \_\_mul\_\_() (simtk.openmm.vec3.Vec3 method)  \_\_ne\_\_ (simtk.openmm.vec3.Vec3 attribute)  \_\_radd\_\_() (simtk.openmm.vec3.Vec3 method)  \_\_reduce\_\_() (simtk.openmm.amd.AMDForceGroupIntegrator method)  (simtk.openmm.amd.AMDIntegrator method)  (simtk.openmm.amd.DualAMDIntegrator method)  (simtk.openmm.app.amberinpcrdfile.AmberInpcrdFile method)  (simtk.openmm.app.amberprmtopfile.AmberPrmtopFile method)  (simtk.openmm.app.charmmcrdfiles.CharmmCrdFile method)  (simtk.openmm.app.charmmcrdfiles.CharmmRstFile method)  (simtk.openmm.app.charmmparameterset.CharmmParameterSet method)  (simtk.openmm.app.charmmpsffile.CharmmPsfFile method)  (simtk.openmm.app.checkpointreporter.CheckpointReporter method)  (simtk.openmm.app.dcdfile.DCDFile method)  (simtk.openmm.app.dcdreporter.DCDReporter method)  (simtk.openmm.app.desmonddmsfile.DesmondDMSFile method)  (simtk.openmm.app.element.Element method)  (simtk.openmm.app.forcefield.ForceField method)  (simtk.openmm.app.gromacsgrofile.GromacsGroFile method)  (simtk.openmm.app.gromacstopfile.GromacsTopFile method)  (simtk.openmm.app.modeller.Modeller method)  (simtk.openmm.app.pdbfile.PDBFile method)  (simtk.openmm.app.pdbreporter.PDBReporter method)  (simtk.openmm.app.pdbreporter.PDBxReporter method)  (simtk.openmm.app.pdbxfile.PDBxFile method)  (simtk.openmm.app.simulation.Simulation method)  (simtk.openmm.app.statedatareporter.StateDataReporter method)  (simtk.openmm.app.topology.Atom method)  (simtk.openmm.app.topology.Chain method)  (simtk.openmm.app.topology.Residue method)  (simtk.openmm.app.topology.Topology method)  (simtk.openmm.mtsintegrator.MTSIntegrator method)  (simtk.openmm.openmm.AmoebaAngleForce method)  (simtk.openmm.openmm.AmoebaBondForce method)  (simtk.openmm.openmm.AmoebaGeneralizedKirkwoodForce method)  (simtk.openmm.openmm.AmoebaInPlaneAngleForce method)  (simtk.openmm.openmm.AmoebaMultipoleForce method)  (simtk.openmm.openmm.AmoebaOutOfPlaneBendForce method)  (simtk.openmm.openmm.AmoebaPiTorsionForce method)  (simtk.openmm.openmm.AmoebaStretchBendForce method)  (simtk.openmm.openmm.AmoebaTorsionTorsionForce method)  (simtk.openmm.openmm.AmoebaVdwForce method)  (simtk.openmm.openmm.AmoebaWcaDispersionForce method)  (simtk.openmm.openmm.AndersenThermostat method)  (simtk.openmm.openmm.BrownianIntegrator method)  (simtk.openmm.openmm.CMAPTorsionForce method)  (simtk.openmm.openmm.CMMotionRemover method)  (simtk.openmm.openmm.CompoundIntegrator method)  (simtk.openmm.openmm.Context method)  (simtk.openmm.openmm.Continuous1DFunction method)  (simtk.openmm.openmm.Continuous2DFunction method)  (simtk.openmm.openmm.Continuous3DFunction method)  (simtk.openmm.openmm.CustomAngleForce method)  (simtk.openmm.openmm.CustomBondForce method)  (simtk.openmm.openmm.CustomCentroidBondForce method)  (simtk.openmm.openmm.CustomCompoundBondForce method)  (simtk.openmm.openmm.CustomExternalForce method)  (simtk.openmm.openmm.CustomGBForce method)  (simtk.openmm.openmm.CustomHbondForce method)  (simtk.openmm.openmm.CustomIntegrator method)  (simtk.openmm.openmm.CustomManyParticleForce method)  (simtk.openmm.openmm.CustomNonbondedForce method)  (simtk.openmm.openmm.CustomTorsionForce method)  (simtk.openmm.openmm.Discrete1DFunction method)  (simtk.openmm.openmm.Discrete2DFunction method)  (simtk.openmm.openmm.Discrete3DFunction method)  (simtk.openmm.openmm.DrudeForce method)  (simtk.openmm.openmm.DrudeLangevinIntegrator method)  (simtk.openmm.openmm.DrudeSCFIntegrator method)  (simtk.openmm.openmm.Force method)  (simtk.openmm.openmm.GBSAOBCForce method)  (simtk.openmm.openmm.HarmonicAngleForce method)  (simtk.openmm.openmm.HarmonicBondForce method)  (simtk.openmm.openmm.Integrator method)  (simtk.openmm.openmm.LangevinIntegrator method)  (simtk.openmm.openmm.LocalCoordinatesSite method)  (simtk.openmm.openmm.LocalEnergyMinimizer method)  (simtk.openmm.openmm.MonteCarloAnisotropicBarostat method)  (simtk.openmm.openmm.MonteCarloBarostat method)  (simtk.openmm.openmm.MonteCarloMembraneBarostat method)  (simtk.openmm.openmm.NonbondedForce method)  (simtk.openmm.openmm.OutOfPlaneSite method)  (simtk.openmm.openmm.PeriodicTorsionForce method)  (simtk.openmm.openmm.Platform method)  (simtk.openmm.openmm.RBTorsionForce method)  (simtk.openmm.openmm.RPMDIntegrator method)  (simtk.openmm.openmm.RPMDMonteCarloBarostat method)  (simtk.openmm.openmm.SerializationNode method)  (simtk.openmm.openmm.SerializationProxy method)  (simtk.openmm.openmm.State method)  (simtk.openmm.openmm.System method)  (simtk.openmm.openmm.TabulatedFunction method)  (simtk.openmm.openmm.ThreeParticleAverageSite method)  (simtk.openmm.openmm.TwoParticleAverageSite method)  (simtk.openmm.openmm.VariableLangevinIntegrator method)  (simtk.openmm.openmm.VariableVerletIntegrator method)  (simtk.openmm.openmm.VerletIntegrator method)  (simtk.openmm.openmm.VirtualSite method)  (simtk.openmm.openmm.XmlSerializer method)  (simtk.openmm.vec3.Vec3 method)  \_\_reduce\_ex\_\_() (simtk.openmm.amd.AMDForceGroupIntegrator method)  (simtk.openmm.amd.AMDIntegrator method)  (simtk.openmm.amd.DualAMDIntegrator method)  (simtk.openmm.app.amberinpcrdfile.AmberInpcrdFile method)  (simtk.openmm.app.amberprmtopfile.AmberPrmtopFile method)  (simtk.openmm.app.charmmcrdfiles.CharmmCrdFile method)  (simtk.openmm.app.charmmcrdfiles.CharmmRstFile method)  (simtk.openmm.app.charmmparameterset.CharmmParameterSet method)  (simtk.openmm.app.charmmpsffile.CharmmPsfFile method)  (simtk.openmm.app.checkpointreporter.CheckpointReporter method)  (simtk.openmm.app.dcdfile.DCDFile method)  (simtk.openmm.app.dcdreporter.DCDReporter method)  (simtk.openmm.app.desmonddmsfile.DesmondDMSFile method)  (simtk.openmm.app.element.Element method)  (simtk.openmm.app.forcefield.ForceField method)  (simtk.openmm.app.gromacsgrofile.GromacsGroFile method)  (simtk.openmm.app.gromacstopfile.GromacsTopFile method)  (simtk.openmm.app.internal.charmm.exceptions.CharmmPSFWarning method)  (simtk.openmm.app.modeller.Modeller method)  (simtk.openmm.app.pdbfile.PDBFile method)  (simtk.openmm.app.pdbreporter.PDBReporter method)  (simtk.openmm.app.pdbreporter.PDBxReporter method)  (simtk.openmm.app.pdbxfile.PDBxFile method)  (simtk.openmm.app.simulation.Simulation method)  (simtk.openmm.app.statedatareporter.StateDataReporter method)  (simtk.openmm.app.topology.Atom method)  (simtk.openmm.app.topology.Chain method)  (simtk.openmm.app.topology.Residue method)  (simtk.openmm.app.topology.Topology method)  (simtk.openmm.mtsintegrator.MTSIntegrator method)  (simtk.openmm.openmm.AmoebaAngleForce method)  (simtk.openmm.openmm.AmoebaBondForce method)  (simtk.openmm.openmm.AmoebaGeneralizedKirkwoodForce method)  (simtk.openmm.openmm.AmoebaInPlaneAngleForce method)  (simtk.openmm.openmm.AmoebaMultipoleForce method)  (simtk.openmm.openmm.AmoebaOutOfPlaneBendForce method)  (simtk.openmm.openmm.AmoebaPiTorsionForce method)  (simtk.openmm.openmm.AmoebaStretchBendForce method)  (simtk.openmm.openmm.AmoebaTorsionTorsionForce method)  (simtk.openmm.openmm.AmoebaVdwForce method)  (simtk.openmm.openmm.AmoebaWcaDispersionForce method)  (simtk.openmm.openmm.AndersenThermostat method)  (simtk.openmm.openmm.BrownianIntegrator method)  (simtk.openmm.openmm.CMAPTorsionForce method)  (simtk.openmm.openmm.CMMotionRemover method)  (simtk.openmm.openmm.CompoundIntegrator method)  (simtk.openmm.openmm.Context method)  (simtk.openmm.openmm.Continuous1DFunction method)  (simtk.openmm.openmm.Continuous2DFunction method)  (simtk.openmm.openmm.Continuous3DFunction method)  (simtk.openmm.openmm.CustomAngleForce method)  (simtk.openmm.openmm.CustomBondForce method)  (simtk.openmm.openmm.CustomCentroidBondForce method)  (simtk.openmm.openmm.CustomCompoundBondForce method)  (simtk.openmm.openmm.CustomExternalForce method)  (simtk.openmm.openmm.CustomGBForce method)  (simtk.openmm.openmm.CustomHbondForce method)  (simtk.openmm.openmm.CustomIntegrator method)  (simtk.openmm.openmm.CustomManyParticleForce method)  (simtk.openmm.openmm.CustomNonbondedForce method)  (simtk.openmm.openmm.CustomTorsionForce method)  (simtk.openmm.openmm.Discrete1DFunction method)  (simtk.openmm.openmm.Discrete2DFunction method)  (simtk.openmm.openmm.Discrete3DFunction method)  (simtk.openmm.openmm.DrudeForce method)  (simtk.openmm.openmm.DrudeLangevinIntegrator method)  (simtk.openmm.openmm.DrudeSCFIntegrator method)  (simtk.openmm.openmm.Force method)  (simtk.openmm.openmm.GBSAOBCForce method)  (simtk.openmm.openmm.HarmonicAngleForce method)  (simtk.openmm.openmm.HarmonicBondForce method)  (simtk.openmm.openmm.Integrator method)  (simtk.openmm.openmm.LangevinIntegrator method)  (simtk.openmm.openmm.LocalCoordinatesSite method)  (simtk.openmm.openmm.LocalEnergyMinimizer method)  (simtk.openmm.openmm.MonteCarloAnisotropicBarostat method)  (simtk.openmm.openmm.MonteCarloBarostat method)  (simtk.openmm.openmm.MonteCarloMembraneBarostat method)  (simtk.openmm.openmm.NonbondedForce method)  (simtk.openmm.openmm.OutOfPlaneSite method)  (simtk.openmm.openmm.PeriodicTorsionForce method)  (simtk.openmm.openmm.Platform method)  (simtk.openmm.openmm.RBTorsionForce method)  (simtk.openmm.openmm.RPMDIntegrator method)  (simtk.openmm.openmm.RPMDMonteCarloBarostat method)  (simtk.openmm.openmm.SerializationNode method)  (simtk.openmm.openmm.SerializationProxy method)  (simtk.openmm.openmm.State method)  (simtk.openmm.openmm.System method)  (simtk.openmm.openmm.TabulatedFunction method)  (simtk.openmm.openmm.ThreeParticleAverageSite method)  (simtk.openmm.openmm.TwoParticleAverageSite method)  (simtk.openmm.openmm.VariableLangevinIntegrator method)  (simtk.openmm.openmm.VariableVerletIntegrator method)  (simtk.openmm.openmm.VerletIntegrator method)  (simtk.openmm.openmm.VirtualSite method)  (simtk.openmm.openmm.XmlSerializer method)  (simtk.openmm.vec3.Vec3 method)  \_\_repr\_\_ (simtk.openmm.app.amberinpcrdfile.AmberInpcrdFile attribute)  (simtk.openmm.app.amberprmtopfile.AmberPrmtopFile attribute)  (simtk.openmm.app.charmmcrdfiles.CharmmCrdFile attribute)  (simtk.openmm.app.charmmcrdfiles.CharmmRstFile attribute)  (simtk.openmm.app.charmmparameterset.CharmmParameterSet attribute)  (simtk.openmm.app.charmmpsffile.CharmmPsfFile attribute)  (simtk.openmm.app.checkpointreporter.CheckpointReporter attribute)  (simtk.openmm.app.dcdfile.DCDFile attribute)  (simtk.openmm.app.dcdreporter.DCDReporter attribute)  (simtk.openmm.app.desmonddmsfile.DesmondDMSFile attribute)  (simtk.openmm.app.forcefield.ForceField attribute)  (simtk.openmm.app.gromacsgrofile.GromacsGroFile attribute)  (simtk.openmm.app.gromacstopfile.GromacsTopFile attribute)  (simtk.openmm.app.internal.charmm.exceptions.CharmmPSFWarning attribute)  (simtk.openmm.app.modeller.Modeller attribute)  (simtk.openmm.app.pdbfile.PDBFile attribute)  (simtk.openmm.app.pdbreporter.PDBReporter attribute)  (simtk.openmm.app.pdbreporter.PDBxReporter attribute)  (simtk.openmm.app.pdbxfile.PDBxFile attribute)  (simtk.openmm.app.simulation.Simulation attribute)  (simtk.openmm.app.statedatareporter.StateDataReporter attribute)  (simtk.openmm.openmm.State attribute)  (simtk.openmm.vec3.Vec3 attribute)  \_\_rmul\_\_() (simtk.openmm.vec3.Vec3 method)  \_\_rsub\_\_() (simtk.openmm.vec3.Vec3 method)  \_\_setattr\_\_ (simtk.openmm.app.amberinpcrdfile.AmberInpcrdFile attribute)  (simtk.openmm.app.amberprmtopfile.AmberPrmtopFile attribute)  (simtk.openmm.app.charmmcrdfiles.CharmmCrdFile attribute)  (simtk.openmm.app.charmmcrdfiles.CharmmRstFile attribute)  (simtk.openmm.app.charmmparameterset.CharmmParameterSet attribute)  (simtk.openmm.app.charmmpsffile.CharmmPsfFile attribute)  (simtk.openmm.app.checkpointreporter.CheckpointReporter attribute)  (simtk.openmm.app.dcdfile.DCDFile attribute)  (simtk.openmm.app.dcdreporter.DCDReporter attribute)  (simtk.openmm.app.desmonddmsfile.DesmondDMSFile attribute)  (simtk.openmm.app.element.Element attribute)  (simtk.openmm.app.forcefield.ForceField attribute)  (simtk.openmm.app.gromacsgrofile.GromacsGroFile attribute)  (simtk.openmm.app.gromacstopfile.GromacsTopFile attribute)  (simtk.openmm.app.internal.charmm.exceptions.CharmmPSFWarning attribute)  (simtk.openmm.app.modeller.Modeller attribute)  (simtk.openmm.app.pdbfile.PDBFile attribute)  (simtk.openmm.app.pdbreporter.PDBReporter attribute)  (simtk.openmm.app.pdbreporter.PDBxReporter attribute)  (simtk.openmm.app.pdbxfile.PDBxFile attribute)  (simtk.openmm.app.simulation.Simulation attribute)  (simtk.openmm.app.statedatareporter.StateDataReporter attribute)  (simtk.openmm.app.topology.Atom attribute)  (simtk.openmm.app.topology.Chain attribute)  (simtk.openmm.app.topology.Residue attribute)  (simtk.openmm.app.topology.Topology attribute)  (simtk.openmm.openmm.State attribute)  (simtk.openmm.vec3.Vec3 attribute)  \_\_sizeof\_\_() (simtk.openmm.amd.AMDForceGroupIntegrator method)  (simtk.openmm.amd.AMDIntegrator method)  (simtk.openmm.amd.DualAMDIntegrator method)  (simtk.openmm.app.amberinpcrdfile.AmberInpcrdFile method)  (simtk.openmm.app.amberprmtopfile.AmberPrmtopFile method)  (simtk.openmm.app.charmmcrdfiles.CharmmCrdFile method)  (simtk.openmm.app.charmmcrdfiles.CharmmRstFile method)  (simtk.openmm.app.charmmparameterset.CharmmParameterSet method)  (simtk.openmm.app.charmmpsffile.CharmmPsfFile method)  (simtk.openmm.app.checkpointreporter.CheckpointReporter method)  (simtk.openmm.app.dcdfile.DCDFile method)  (simtk.openmm.app.dcdreporter.DCDReporter method)  (simtk.openmm.app.desmonddmsfile.DesmondDMSFile method)  (simtk.openmm.app.element.Element method)  (simtk.openmm.app.forcefield.ForceField method)  (simtk.openmm.app.gromacsgrofile.GromacsGroFile method)  (simtk.openmm.app.gromacstopfile.GromacsTopFile method)  (simtk.openmm.app.internal.charmm.exceptions.CharmmPSFWarning method)  (simtk.openmm.app.modeller.Modeller method)  (simtk.openmm.app.pdbfile.PDBFile method)  (simtk.openmm.app.pdbreporter.PDBReporter method)  (simtk.openmm.app.pdbreporter.PDBxReporter method)  (simtk.openmm.app.pdbxfile.PDBxFile method)  (simtk.openmm.app.simulation.Simulation method)  (simtk.openmm.app.statedatareporter.StateDataReporter method)  (simtk.openmm.app.topology.Atom method)  (simtk.openmm.app.topology.Chain method)  (simtk.openmm.app.topology.Residue method)  (simtk.openmm.app.topology.Topology method)  (simtk.openmm.mtsintegrator.MTSIntegrator method)  (simtk.openmm.openmm.AmoebaAngleForce method)  (simtk.openmm.openmm.AmoebaBondForce method)  (simtk.openmm.openmm.AmoebaGeneralizedKirkwoodForce method)  (simtk.openmm.openmm.AmoebaInPlaneAngleForce method)  (simtk.openmm.openmm.AmoebaMultipoleForce method)  (simtk.openmm.openmm.AmoebaOutOfPlaneBendForce method)  (simtk.openmm.openmm.AmoebaPiTorsionForce method)  (simtk.openmm.openmm.AmoebaStretchBendForce method)  (simtk.openmm.openmm.AmoebaTorsionTorsionForce method)  (simtk.openmm.openmm.AmoebaVdwForce method)  (simtk.openmm.openmm.AmoebaWcaDispersionForce method)  (simtk.openmm.openmm.AndersenThermostat method)  (simtk.openmm.openmm.BrownianIntegrator method)  (simtk.openmm.openmm.CMAPTorsionForce method)  (simtk.openmm.openmm.CMMotionRemover method)  (simtk.openmm.openmm.CompoundIntegrator method)  (simtk.openmm.openmm.Context method)  (simtk.openmm.openmm.Continuous1DFunction method)  (simtk.openmm.openmm.Continuous2DFunction method)  (simtk.openmm.openmm.Continuous3DFunction method)  (simtk.openmm.openmm.CustomAngleForce method)  (simtk.openmm.openmm.CustomBondForce method)  (simtk.openmm.openmm.CustomCentroidBondForce method)  (simtk.openmm.openmm.CustomCompoundBondForce method)  (simtk.openmm.openmm.CustomExternalForce method)  (simtk.openmm.openmm.CustomGBForce method)  (simtk.openmm.openmm.CustomHbondForce method)  (simtk.openmm.openmm.CustomIntegrator method)  (simtk.openmm.openmm.CustomManyParticleForce method)  (simtk.openmm.openmm.CustomNonbondedForce method)  (simtk.openmm.openmm.CustomTorsionForce method)  (simtk.openmm.openmm.Discrete1DFunction method)  (simtk.openmm.openmm.Discrete2DFunction method)  (simtk.openmm.openmm.Discrete3DFunction method)  (simtk.openmm.openmm.DrudeForce method)  (simtk.openmm.openmm.DrudeLangevinIntegrator method)  (simtk.openmm.openmm.DrudeSCFIntegrator method)  (simtk.openmm.openmm.Force method)  (simtk.openmm.openmm.GBSAOBCForce method)  (simtk.openmm.openmm.HarmonicAngleForce method)  (simtk.openmm.openmm.HarmonicBondForce method)  (simtk.openmm.openmm.Integrator method)  (simtk.openmm.openmm.LangevinIntegrator method)  (simtk.openmm.openmm.LocalCoordinatesSite method)  (simtk.openmm.openmm.LocalEnergyMinimizer method)  (simtk.openmm.openmm.MonteCarloAnisotropicBarostat method)  (simtk.openmm.openmm.MonteCarloBarostat method)  (simtk.openmm.openmm.MonteCarloMembraneBarostat method)  (simtk.openmm.openmm.NonbondedForce method)  (simtk.openmm.openmm.OutOfPlaneSite method)  (simtk.openmm.openmm.PeriodicTorsionForce method)  (simtk.openmm.openmm.Platform method)  (simtk.openmm.openmm.RBTorsionForce method)  (simtk.openmm.openmm.RPMDIntegrator method)  (simtk.openmm.openmm.RPMDMonteCarloBarostat method)  (simtk.openmm.openmm.SerializationNode method)  (simtk.openmm.openmm.SerializationProxy method)  (simtk.openmm.openmm.State method)  (simtk.openmm.openmm.System method)  (simtk.openmm.openmm.TabulatedFunction method)  (simtk.openmm.openmm.ThreeParticleAverageSite method)  (simtk.openmm.openmm.TwoParticleAverageSite method)  (simtk.openmm.openmm.VariableLangevinIntegrator method)  (simtk.openmm.openmm.VariableVerletIntegrator method)  (simtk.openmm.openmm.VerletIntegrator method)  (simtk.openmm.openmm.VirtualSite method)  (simtk.openmm.openmm.XmlSerializer method)  (simtk.openmm.vec3.Vec3 method)  \_\_str\_\_ (simtk.openmm.amd.AMDForceGroupIntegrator attribute)  (simtk.openmm.amd.AMDIntegrator attribute)  (simtk.openmm.amd.DualAMDIntegrator attribute)  (simtk.openmm.app.amberinpcrdfile.AmberInpcrdFile attribute)  (simtk.openmm.app.amberprmtopfile.AmberPrmtopFile attribute)  (simtk.openmm.app.charmmcrdfiles.CharmmCrdFile attribute)  (simtk.openmm.app.charmmcrdfiles.CharmmRstFile attribute)  (simtk.openmm.app.charmmparameterset.CharmmParameterSet attribute)  (simtk.openmm.app.charmmpsffile.CharmmPsfFile attribute)  (simtk.openmm.app.checkpointreporter.CheckpointReporter attribute)  (simtk.openmm.app.dcdfile.DCDFile attribute)  (simtk.openmm.app.dcdreporter.DCDReporter attribute)  (simtk.openmm.app.desmonddmsfile.DesmondDMSFile attribute)  (simtk.openmm.app.forcefield.ForceField attribute)  (simtk.openmm.app.gromacsgrofile.GromacsGroFile attribute)  (simtk.openmm.app.gromacstopfile.GromacsTopFile attribute)  (simtk.openmm.app.internal.charmm.exceptions.CharmmPSFWarning attribute)  (simtk.openmm.app.modeller.Modeller attribute)  (simtk.openmm.app.pdbfile.PDBFile attribute)  (simtk.openmm.app.pdbreporter.PDBReporter attribute)  (simtk.openmm.app.pdbreporter.PDBxReporter attribute)  (simtk.openmm.app.pdbxfile.PDBxFile attribute)  (simtk.openmm.app.simulation.Simulation attribute)  (simtk.openmm.app.statedatareporter.StateDataReporter attribute)  (simtk.openmm.app.topology.Atom attribute)  (simtk.openmm.app.topology.Chain attribute)  (simtk.openmm.app.topology.Residue attribute)  (simtk.openmm.app.topology.Topology attribute)  (simtk.openmm.mtsintegrator.MTSIntegrator attribute)  (simtk.openmm.openmm.AmoebaAngleForce attribute)  (simtk.openmm.openmm.AmoebaBondForce attribute)  (simtk.openmm.openmm.AmoebaGeneralizedKirkwoodForce attribute)  (simtk.openmm.openmm.AmoebaInPlaneAngleForce attribute)  (simtk.openmm.openmm.AmoebaMultipoleForce attribute)  (simtk.openmm.openmm.AmoebaOutOfPlaneBendForce attribute)  (simtk.openmm.openmm.AmoebaPiTorsionForce attribute)  (simtk.openmm.openmm.AmoebaStretchBendForce attribute)  (simtk.openmm.openmm.AmoebaTorsionTorsionForce attribute)  (simtk.openmm.openmm.AmoebaVdwForce attribute)  (simtk.openmm.openmm.AmoebaWcaDispersionForce attribute)  (simtk.openmm.openmm.AndersenThermostat attribute)  (simtk.openmm.openmm.BrownianIntegrator attribute)  (simtk.openmm.openmm.CMAPTorsionForce attribute)  (simtk.openmm.openmm.CMMotionRemover attribute)  (simtk.openmm.openmm.CompoundIntegrator attribute)  (simtk.openmm.openmm.Context attribute)  (simtk.openmm.openmm.Continuous1DFunction attribute)  (simtk.openmm.openmm.Continuous2DFunction attribute)  (simtk.openmm.openmm.Continuous3DFunction attribute)  (simtk.openmm.openmm.CustomAngleForce attribute)  (simtk.openmm.openmm.CustomBondForce attribute)  (simtk.openmm.openmm.CustomCentroidBondForce attribute)  (simtk.openmm.openmm.CustomCompoundBondForce attribute)  (simtk.openmm.openmm.CustomExternalForce attribute)  (simtk.openmm.openmm.CustomGBForce attribute)  (simtk.openmm.openmm.CustomHbondForce attribute)  (simtk.openmm.openmm.CustomIntegrator attribute)  (simtk.openmm.openmm.CustomManyParticleForce attribute)  (simtk.openmm.openmm.CustomNonbondedForce attribute)  (simtk.openmm.openmm.CustomTorsionForce attribute)  (simtk.openmm.openmm.Discrete1DFunction attribute)  (simtk.openmm.openmm.Discrete2DFunction attribute)  (simtk.openmm.openmm.Discrete3DFunction attribute)  (simtk.openmm.openmm.DrudeForce attribute)  (simtk.openmm.openmm.DrudeLangevinIntegrator attribute)  (simtk.openmm.openmm.DrudeSCFIntegrator attribute)  (simtk.openmm.openmm.Force attribute)  (simtk.openmm.openmm.GBSAOBCForce attribute)  (simtk.openmm.openmm.HarmonicAngleForce attribute)  (simtk.openmm.openmm.HarmonicBondForce attribute)  (simtk.openmm.openmm.Integrator attribute)  (simtk.openmm.openmm.LangevinIntegrator attribute)  (simtk.openmm.openmm.LocalCoordinatesSite attribute)  (simtk.openmm.openmm.LocalEnergyMinimizer attribute)  (simtk.openmm.openmm.MonteCarloAnisotropicBarostat attribute)  (simtk.openmm.openmm.MonteCarloBarostat attribute)  (simtk.openmm.openmm.MonteCarloMembraneBarostat attribute)  (simtk.openmm.openmm.NonbondedForce attribute)  (simtk.openmm.openmm.OutOfPlaneSite attribute)  (simtk.openmm.openmm.PeriodicTorsionForce attribute)  (simtk.openmm.openmm.Platform attribute)  (simtk.openmm.openmm.RBTorsionForce attribute)  (simtk.openmm.openmm.RPMDIntegrator attribute)  (simtk.openmm.openmm.RPMDMonteCarloBarostat attribute)  (simtk.openmm.openmm.SerializationNode attribute)  (simtk.openmm.openmm.SerializationProxy attribute)  (simtk.openmm.openmm.State attribute)  (simtk.openmm.openmm.System attribute)  (simtk.openmm.openmm.TabulatedFunction attribute)  (simtk.openmm.openmm.ThreeParticleAverageSite attribute)  (simtk.openmm.openmm.TwoParticleAverageSite attribute)  (simtk.openmm.openmm.VariableLangevinIntegrator attribute)  (simtk.openmm.openmm.VariableVerletIntegrator attribute)  (simtk.openmm.openmm.VerletIntegrator attribute)  (simtk.openmm.openmm.VirtualSite attribute)  (simtk.openmm.openmm.XmlSerializer attribute)  (simtk.openmm.vec3.Vec3 attribute)  \_\_sub\_\_() (simtk.openmm.vec3.Vec3 method)  \_\_truediv\_\_() (simtk.openmm.vec3.Vec3 method) |

## A

|  |  |
| --- | --- |
| add() (simtk.openmm.app.modeller.Modeller method)  addAcceptor() (simtk.openmm.openmm.CustomHbondForce method)  addAngle() (simtk.openmm.openmm.AmoebaAngleForce method)  (simtk.openmm.openmm.AmoebaInPlaneAngleForce method)  (simtk.openmm.openmm.CustomAngleForce method)  (simtk.openmm.openmm.HarmonicAngleForce method)  addAtom() (simtk.openmm.app.topology.Topology method)  addBond() (simtk.openmm.app.topology.Topology method)  (simtk.openmm.openmm.AmoebaBondForce method)  (simtk.openmm.openmm.CustomBondForce method)  (simtk.openmm.openmm.CustomCentroidBondForce method)  (simtk.openmm.openmm.CustomCompoundBondForce method)  (simtk.openmm.openmm.HarmonicBondForce method)  addChain() (simtk.openmm.app.topology.Topology method)  addComputedValue() (simtk.openmm.openmm.CustomGBForce method)  addComputeGlobal() (simtk.openmm.amd.AMDForceGroupIntegrator method)  (simtk.openmm.amd.AMDIntegrator method)  (simtk.openmm.amd.DualAMDIntegrator method)  (simtk.openmm.mtsintegrator.MTSIntegrator method)  (simtk.openmm.openmm.CustomIntegrator method)  addComputePerDof() (simtk.openmm.amd.AMDForceGroupIntegrator method)  (simtk.openmm.amd.AMDIntegrator method)  (simtk.openmm.amd.DualAMDIntegrator method)  (simtk.openmm.mtsintegrator.MTSIntegrator method)  (simtk.openmm.openmm.CustomIntegrator method)  addComputeSum() (simtk.openmm.amd.AMDForceGroupIntegrator method)  (simtk.openmm.amd.AMDIntegrator method)  (simtk.openmm.amd.DualAMDIntegrator method)  (simtk.openmm.mtsintegrator.MTSIntegrator method)  (simtk.openmm.openmm.CustomIntegrator method)  addConstrainPositions() (simtk.openmm.amd.AMDForceGroupIntegrator method)  (simtk.openmm.amd.AMDIntegrator method)  (simtk.openmm.amd.DualAMDIntegrator method)  (simtk.openmm.mtsintegrator.MTSIntegrator method)  (simtk.openmm.openmm.CustomIntegrator method)  addConstraint() (simtk.openmm.openmm.System method)  addConstrainVelocities() (simtk.openmm.amd.AMDForceGroupIntegrator method)  (simtk.openmm.amd.AMDIntegrator method)  (simtk.openmm.amd.DualAMDIntegrator method)  (simtk.openmm.mtsintegrator.MTSIntegrator method)  (simtk.openmm.openmm.CustomIntegrator method)  addDonor() (simtk.openmm.openmm.CustomHbondForce method)  addEnergyTerm() (simtk.openmm.openmm.CustomGBForce method)  addException() (simtk.openmm.openmm.NonbondedForce method)  addException\_usingRMin() (simtk.openmm.openmm.NonbondedForce method)  addExclusion() (simtk.openmm.openmm.CustomGBForce method)  (simtk.openmm.openmm.CustomHbondForce method)  (simtk.openmm.openmm.CustomManyParticleForce method)  (simtk.openmm.openmm.CustomNonbondedForce method)  addExtraParticles() (simtk.openmm.app.modeller.Modeller method)  addForce() (simtk.openmm.openmm.System method)  addFunction() (simtk.openmm.openmm.CustomCompoundBondForce method)  (simtk.openmm.openmm.CustomGBForce method)  (simtk.openmm.openmm.CustomHbondForce method)  (simtk.openmm.openmm.CustomNonbondedForce method)  addGlobalParameter() (simtk.openmm.openmm.CustomAngleForce method)  (simtk.openmm.openmm.CustomBondForce method)  (simtk.openmm.openmm.CustomCentroidBondForce method)  (simtk.openmm.openmm.CustomCompoundBondForce method)  (simtk.openmm.openmm.CustomExternalForce method)  (simtk.openmm.openmm.CustomGBForce method)  (simtk.openmm.openmm.CustomHbondForce method)  (simtk.openmm.openmm.CustomManyParticleForce method)  (simtk.openmm.openmm.CustomNonbondedForce method)  (simtk.openmm.openmm.CustomTorsionForce method)  addGlobalVariable() (simtk.openmm.amd.AMDForceGroupIntegrator method)  (simtk.openmm.amd.AMDIntegrator method)  (simtk.openmm.amd.DualAMDIntegrator method)  (simtk.openmm.mtsintegrator.MTSIntegrator method)  (simtk.openmm.openmm.CustomIntegrator method)  addGroup() (simtk.openmm.openmm.CustomCentroidBondForce method)  addHydrogens() (simtk.openmm.app.modeller.Modeller method)  addIntegrator() (simtk.openmm.openmm.CompoundIntegrator method)  addInteractionGroup() (simtk.openmm.openmm.CustomNonbondedForce method)  addMap() (simtk.openmm.openmm.CMAPTorsionForce method)  addMultipole() (simtk.openmm.openmm.AmoebaMultipoleForce method)  addOutOfPlaneBend() (simtk.openmm.openmm.AmoebaOutOfPlaneBendForce method)  addParticle() (simtk.openmm.openmm.AmoebaGeneralizedKirkwoodForce method)  (simtk.openmm.openmm.AmoebaVdwForce method)  (simtk.openmm.openmm.AmoebaWcaDispersionForce method)  (simtk.openmm.openmm.CustomExternalForce method)  (simtk.openmm.openmm.CustomGBForce method)  (simtk.openmm.openmm.CustomManyParticleForce method)  (simtk.openmm.openmm.CustomNonbondedForce method)  (simtk.openmm.openmm.DrudeForce method)  (simtk.openmm.openmm.GBSAOBCForce method)  (simtk.openmm.openmm.NonbondedForce method)  (simtk.openmm.openmm.System method)  addParticle\_usingRVdw() (simtk.openmm.openmm.NonbondedForce method)  addPerAcceptorParameter() (simtk.openmm.openmm.CustomHbondForce method)  addPerAngleParameter() (simtk.openmm.openmm.CustomAngleForce method) | addPerBondParameter() (simtk.openmm.openmm.CustomBondForce method)  (simtk.openmm.openmm.CustomCentroidBondForce method)  (simtk.openmm.openmm.CustomCompoundBondForce method)  addPerDofVariable() (simtk.openmm.amd.AMDForceGroupIntegrator method)  (simtk.openmm.amd.AMDIntegrator method)  (simtk.openmm.amd.DualAMDIntegrator method)  (simtk.openmm.mtsintegrator.MTSIntegrator method)  (simtk.openmm.openmm.CustomIntegrator method)  addPerDonorParameter() (simtk.openmm.openmm.CustomHbondForce method)  addPerParticleParameter() (simtk.openmm.openmm.CustomExternalForce method)  (simtk.openmm.openmm.CustomGBForce method)  (simtk.openmm.openmm.CustomManyParticleForce method)  (simtk.openmm.openmm.CustomNonbondedForce method)  addPerTorsionParameter() (simtk.openmm.openmm.CustomTorsionForce method)  addPiTorsion() (simtk.openmm.openmm.AmoebaPiTorsionForce method)  addResidue() (simtk.openmm.app.topology.Topology method)  addScreenedPair() (simtk.openmm.openmm.DrudeForce method)  addSolvent() (simtk.openmm.app.modeller.Modeller method)  addStretchBend() (simtk.openmm.openmm.AmoebaStretchBendForce method)  addTabulatedFunction() (simtk.openmm.openmm.CustomCentroidBondForce method)  (simtk.openmm.openmm.CustomCompoundBondForce method)  (simtk.openmm.openmm.CustomGBForce method)  (simtk.openmm.openmm.CustomHbondForce method)  (simtk.openmm.openmm.CustomManyParticleForce method)  (simtk.openmm.openmm.CustomNonbondedForce method)  addTorsion() (simtk.openmm.openmm.CMAPTorsionForce method)  (simtk.openmm.openmm.CustomTorsionForce method)  (simtk.openmm.openmm.PeriodicTorsionForce method)  (simtk.openmm.openmm.RBTorsionForce method)  addTorsionTorsion() (simtk.openmm.openmm.AmoebaTorsionTorsionForce method)  addUpdateContextState() (simtk.openmm.amd.AMDForceGroupIntegrator method)  (simtk.openmm.amd.AMDIntegrator method)  (simtk.openmm.amd.DualAMDIntegrator method)  (simtk.openmm.mtsintegrator.MTSIntegrator method)  (simtk.openmm.openmm.CustomIntegrator method)  AmberInpcrdFile (class in simtk.openmm.app.amberinpcrdfile)  AmberPrmtopFile (class in simtk.openmm.app.amberprmtopfile)  AMDForceGroupIntegrator (class in simtk.openmm.amd)  AMDIntegrator (class in simtk.openmm.amd)  AmoebaAngleForce (class in simtk.openmm.openmm)  AmoebaBondForce (class in simtk.openmm.openmm)  AmoebaGeneralizedKirkwoodForce (class in simtk.openmm.openmm)  AmoebaInPlaneAngleForce (class in simtk.openmm.openmm)  AmoebaMultipoleForce (class in simtk.openmm.openmm)  AmoebaOutOfPlaneBendForce (class in simtk.openmm.openmm)  AmoebaPiTorsionForce (class in simtk.openmm.openmm)  AmoebaStretchBendForce (class in simtk.openmm.openmm)  AmoebaTorsionTorsionForce (class in simtk.openmm.openmm)  AmoebaVdwForce (class in simtk.openmm.openmm)  AmoebaWcaDispersionForce (class in simtk.openmm.openmm)  AndersenThermostat (class in simtk.openmm.openmm)  applyConstraints() (simtk.openmm.openmm.Context method)  applyVelocityConstraints() (simtk.openmm.openmm.Context method)  Atom (class in simtk.openmm.app.topology)  atoms() (simtk.openmm.app.topology.Chain method)  (simtk.openmm.app.topology.Residue method)  (simtk.openmm.app.topology.Topology method) |

## B

|  |  |
| --- | --- |
| beginIfBlock() (simtk.openmm.amd.AMDForceGroupIntegrator method)  (simtk.openmm.amd.AMDIntegrator method)  (simtk.openmm.amd.DualAMDIntegrator method)  (simtk.openmm.mtsintegrator.MTSIntegrator method)  (simtk.openmm.openmm.CustomIntegrator method)  beginWhileBlock() (simtk.openmm.amd.AMDForceGroupIntegrator method)  (simtk.openmm.amd.AMDIntegrator method)  (simtk.openmm.amd.DualAMDIntegrator method)  (simtk.openmm.mtsintegrator.MTSIntegrator method)  (simtk.openmm.openmm.CustomIntegrator method)  bonds() (simtk.openmm.app.topology.Residue method)  (simtk.openmm.app.topology.Topology method) | boxLengths (simtk.openmm.app.charmmpsffile.CharmmPsfFile attribute)  boxVectors (simtk.openmm.app.charmmpsffile.CharmmPsfFile attribute)  BrownianIntegrator (class in simtk.openmm.openmm) |

## C

|  |  |
| --- | --- |
| Chain (class in simtk.openmm.app.topology)  chains() (simtk.openmm.app.topology.Topology method)  CharmmCrdFile (class in simtk.openmm.app.charmmcrdfiles)  CharmmParameterSet (class in simtk.openmm.app.charmmparameterset)  CharmmPsfFile (class in simtk.openmm.app.charmmpsffile)  CharmmPSFWarning (class in simtk.openmm.app.internal.charmm.exceptions)  CharmmRstFile (class in simtk.openmm.app.charmmcrdfiles)  CheckpointReporter (class in simtk.openmm.app.checkpointreporter)  close() (simtk.openmm.app.desmonddmsfile.DesmondDMSFile method)  CMAPTorsionForce (class in simtk.openmm.openmm)  CMMotionRemover (class in simtk.openmm.openmm)  CollisionFrequency() (simtk.openmm.openmm.AndersenThermostat static method)  CompoundIntegrator (class in simtk.openmm.openmm)  computeVirtualSites() (simtk.openmm.openmm.Context method)  condense() (simtk.openmm.app.charmmparameterset.CharmmParameterSet method)  Context (class in simtk.openmm.openmm)  Continuous1DFunction (class in simtk.openmm.openmm)  Continuous2DFunction (class in simtk.openmm.openmm)  Continuous3DFunction (class in simtk.openmm.openmm)  convertWater() (simtk.openmm.app.modeller.Modeller method) | Copy() (simtk.openmm.openmm.Continuous1DFunction method)  (simtk.openmm.openmm.Continuous2DFunction method)  (simtk.openmm.openmm.Continuous3DFunction method)  (simtk.openmm.openmm.Discrete1DFunction method)  (simtk.openmm.openmm.Discrete2DFunction method)  (simtk.openmm.openmm.Discrete3DFunction method)  (simtk.openmm.openmm.TabulatedFunction method)  count() (simtk.openmm.vec3.Vec3 method)  createCheckpoint() (simtk.openmm.openmm.Context method)  createChildNode() (simtk.openmm.openmm.SerializationNode method)  createDisulfideBonds() (simtk.openmm.app.topology.Topology method)  createExceptionsFromBonds() (simtk.openmm.openmm.NonbondedForce method)  createExclusionsFromBonds() (simtk.openmm.openmm.CustomManyParticleForce method)  (simtk.openmm.openmm.CustomNonbondedForce method)  createStandardBonds() (simtk.openmm.app.topology.Topology method)  createSystem() (simtk.openmm.app.amberprmtopfile.AmberPrmtopFile method)  (simtk.openmm.app.charmmpsffile.CharmmPsfFile method)  (simtk.openmm.app.desmonddmsfile.DesmondDMSFile method)  (simtk.openmm.app.forcefield.ForceField method)  (simtk.openmm.app.gromacstopfile.GromacsTopFile method)  CustomAngleForce (class in simtk.openmm.openmm)  CustomBondForce (class in simtk.openmm.openmm)  CustomCentroidBondForce (class in simtk.openmm.openmm)  CustomCompoundBondForce (class in simtk.openmm.openmm)  CustomExternalForce (class in simtk.openmm.openmm)  CustomGBForce (class in simtk.openmm.openmm)  CustomHbondForce (class in simtk.openmm.openmm)  CustomIntegrator (class in simtk.openmm.openmm)  CustomManyParticleForce (class in simtk.openmm.openmm)  CustomNonbondedForce (class in simtk.openmm.openmm)  CustomTorsionForce (class in simtk.openmm.openmm) |

## D

|  |  |
| --- | --- |
| DCDFile (class in simtk.openmm.app.dcdfile)  DCDReporter (class in simtk.openmm.app.dcdreporter)  delete() (simtk.openmm.app.modeller.Modeller method)  deleteCmap() (simtk.openmm.app.charmmpsffile.CharmmPsfFile method)  deleteWater() (simtk.openmm.app.modeller.Modeller method)  describeNextReport() (simtk.openmm.app.checkpointreporter.CheckpointReporter method)  (simtk.openmm.app.dcdreporter.DCDReporter method)  (simtk.openmm.app.pdbreporter.PDBReporter method)  (simtk.openmm.app.pdbreporter.PDBxReporter method)  (simtk.openmm.app.statedatareporter.StateDataReporter method)  deserialize() (simtk.openmm.openmm.SerializationProxy method)  (simtk.openmm.openmm.XmlSerializer static method)  deserializeSystem() (simtk.openmm.openmm.XmlSerializer static method) | DesmondDMSFile (class in simtk.openmm.app.desmonddmsfile)  Discrete1DFunction (class in simtk.openmm.openmm)  Discrete2DFunction (class in simtk.openmm.openmm)  Discrete3DFunction (class in simtk.openmm.openmm)  DrudeForce (class in simtk.openmm.openmm)  DrudeLangevinIntegrator (class in simtk.openmm.openmm)  DrudeSCFIntegrator (class in simtk.openmm.openmm)  DualAMDIntegrator (class in simtk.openmm.amd) |

## E

|  |  |
| --- | --- |
| Element (class in simtk.openmm.app.element)  endBlock() (simtk.openmm.amd.AMDForceGroupIntegrator method)  (simtk.openmm.amd.AMDIntegrator method)  (simtk.openmm.amd.DualAMDIntegrator method)  (simtk.openmm.mtsintegrator.MTSIntegrator method)  (simtk.openmm.openmm.CustomIntegrator method) | external\_bonds() (simtk.openmm.app.topology.Residue method) |

## F

|  |  |
| --- | --- |
| findPlatform() (simtk.openmm.openmm.Platform static method)  Force (class in simtk.openmm.openmm) | ForceField (class in simtk.openmm.app.forcefield) |

## G

|  |  |
| --- | --- |
| GBSAOBCForce (class in simtk.openmm.openmm)  generateTemplatesForUnmatchedResidues() (simtk.openmm.app.forcefield.ForceField method)  getAcceptorParameters() (simtk.openmm.openmm.CustomHbondForce method)  getAEwald() (simtk.openmm.openmm.AmoebaMultipoleForce method)  getAlpha() (simtk.openmm.amd.AMDIntegrator method)  getAlphaGroup() (simtk.openmm.amd.AMDForceGroupIntegrator method)  (simtk.openmm.amd.DualAMDIntegrator method)  getAlphaTotal() (simtk.openmm.amd.DualAMDIntegrator method)  getAmoebaGlobalAngleCubic() (simtk.openmm.openmm.AmoebaAngleForce method)  getAmoebaGlobalAnglePentic() (simtk.openmm.openmm.AmoebaAngleForce method)  getAmoebaGlobalAngleQuartic() (simtk.openmm.openmm.AmoebaAngleForce method)  getAmoebaGlobalAngleSextic() (simtk.openmm.openmm.AmoebaAngleForce method)  getAmoebaGlobalBondCubic() (simtk.openmm.openmm.AmoebaBondForce method)  getAmoebaGlobalBondQuartic() (simtk.openmm.openmm.AmoebaBondForce method)  getAmoebaGlobalInPlaneAngleCubic() (simtk.openmm.openmm.AmoebaInPlaneAngleForce method)  getAmoebaGlobalInPlaneAnglePentic() (simtk.openmm.openmm.AmoebaInPlaneAngleForce method)  getAmoebaGlobalInPlaneAngleQuartic() (simtk.openmm.openmm.AmoebaInPlaneAngleForce method)  getAmoebaGlobalInPlaneAngleSextic() (simtk.openmm.openmm.AmoebaInPlaneAngleForce method)  getAmoebaGlobalOutOfPlaneBendCubic() (simtk.openmm.openmm.AmoebaOutOfPlaneBendForce method)  getAmoebaGlobalOutOfPlaneBendPentic() (simtk.openmm.openmm.AmoebaOutOfPlaneBendForce method)  getAmoebaGlobalOutOfPlaneBendQuartic() (simtk.openmm.openmm.AmoebaOutOfPlaneBendForce method)  getAmoebaGlobalOutOfPlaneBendSextic() (simtk.openmm.openmm.AmoebaOutOfPlaneBendForce method)  getAngleParameters() (simtk.openmm.openmm.AmoebaAngleForce method)  (simtk.openmm.openmm.AmoebaInPlaneAngleForce method)  (simtk.openmm.openmm.CustomAngleForce method)  (simtk.openmm.openmm.HarmonicAngleForce method)  getApplyThermostat() (simtk.openmm.openmm.RPMDIntegrator method)  getAwater() (simtk.openmm.openmm.AmoebaWcaDispersionForce method)  getBondParameters() (simtk.openmm.openmm.AmoebaBondForce method)  (simtk.openmm.openmm.CustomBondForce method)  (simtk.openmm.openmm.CustomCentroidBondForce method)  (simtk.openmm.openmm.CustomCompoundBondForce method)  (simtk.openmm.openmm.HarmonicBondForce method)  getBoolProperty() (simtk.openmm.openmm.SerializationNode method)  getBoxVectors() (simtk.openmm.app.amberinpcrdfile.AmberInpcrdFile method)  getByMass() (simtk.openmm.app.element.Element static method)  getBySymbol() (simtk.openmm.app.element.Element static method)  getChildNode() (simtk.openmm.openmm.SerializationNode method)  getChildren() (simtk.openmm.openmm.SerializationNode method)  getComputationStep() (simtk.openmm.amd.AMDForceGroupIntegrator method)  (simtk.openmm.amd.AMDIntegrator method)  (simtk.openmm.amd.DualAMDIntegrator method)  (simtk.openmm.mtsintegrator.MTSIntegrator method)  (simtk.openmm.openmm.CustomIntegrator method)  getComputedValueParameters() (simtk.openmm.openmm.CustomGBForce method)  getConstraintParameters() (simtk.openmm.openmm.System method)  getConstraintTolerance() (simtk.openmm.amd.AMDForceGroupIntegrator method)  (simtk.openmm.amd.AMDIntegrator method)  (simtk.openmm.amd.DualAMDIntegrator method)  (simtk.openmm.mtsintegrator.MTSIntegrator method)  (simtk.openmm.openmm.BrownianIntegrator method)  (simtk.openmm.openmm.CompoundIntegrator method)  (simtk.openmm.openmm.CustomIntegrator method)  (simtk.openmm.openmm.DrudeLangevinIntegrator method)  (simtk.openmm.openmm.DrudeSCFIntegrator method)  (simtk.openmm.openmm.Integrator method)  (simtk.openmm.openmm.LangevinIntegrator method)  (simtk.openmm.openmm.RPMDIntegrator method)  (simtk.openmm.openmm.VariableLangevinIntegrator method)  (simtk.openmm.openmm.VariableVerletIntegrator method)  (simtk.openmm.openmm.VerletIntegrator method)  getContractions() (simtk.openmm.openmm.RPMDIntegrator method)  getCovalentMap() (simtk.openmm.openmm.AmoebaMultipoleForce method)  getCovalentMaps() (simtk.openmm.openmm.AmoebaMultipoleForce method)  getCurrentIntegrator() (simtk.openmm.openmm.CompoundIntegrator method)  getCutoff() (simtk.openmm.openmm.AmoebaVdwForce method)  getCutoffDistance() (simtk.openmm.openmm.AmoebaMultipoleForce method)  (simtk.openmm.openmm.CustomGBForce method)  (simtk.openmm.openmm.CustomHbondForce method)  (simtk.openmm.openmm.CustomManyParticleForce method)  (simtk.openmm.openmm.CustomNonbondedForce method)  (simtk.openmm.openmm.GBSAOBCForce method)  (simtk.openmm.openmm.NonbondedForce method)  getDefaultCollisionFrequency() (simtk.openmm.openmm.AndersenThermostat method)  getDefaultPeriodicBoxVectors() (simtk.openmm.openmm.System method)  getDefaultPluginsDirectory() (simtk.openmm.openmm.Platform static method)  getDefaultPressure() (simtk.openmm.openmm.MonteCarloAnisotropicBarostat method)  (simtk.openmm.openmm.MonteCarloBarostat method)  (simtk.openmm.openmm.MonteCarloMembraneBarostat method)  (simtk.openmm.openmm.RPMDMonteCarloBarostat method)  getDefaultSurfaceTension() (simtk.openmm.openmm.MonteCarloMembraneBarostat method)  getDefaultTemperature() (simtk.openmm.openmm.AndersenThermostat method)  getDispoff() (simtk.openmm.openmm.AmoebaWcaDispersionForce method)  getDonorParameters() (simtk.openmm.openmm.CustomHbondForce method)  getDoubleProperty() (simtk.openmm.openmm.SerializationNode method)  getDrudeFriction() (simtk.openmm.openmm.DrudeLangevinIntegrator method)  getDrudeTemperature() (simtk.openmm.openmm.DrudeLangevinIntegrator method)  getE() (simtk.openmm.amd.AMDIntegrator method)  getEffectiveEnergy() (simtk.openmm.amd.AMDForceGroupIntegrator method)  (simtk.openmm.amd.AMDIntegrator method)  (simtk.openmm.amd.DualAMDIntegrator method)  getEGroup() (simtk.openmm.amd.AMDForceGroupIntegrator method)  (simtk.openmm.amd.DualAMDIntegrator method)  getElectrostaticPotential() (simtk.openmm.openmm.AmoebaMultipoleForce method)  getEnergyFunction() (simtk.openmm.openmm.CustomAngleForce method)  (simtk.openmm.openmm.CustomBondForce method)  (simtk.openmm.openmm.CustomCentroidBondForce method)  (simtk.openmm.openmm.CustomCompoundBondForce method)  (simtk.openmm.openmm.CustomExternalForce method)  (simtk.openmm.openmm.CustomHbondForce method)  (simtk.openmm.openmm.CustomManyParticleForce method)  (simtk.openmm.openmm.CustomNonbondedForce method)  (simtk.openmm.openmm.CustomTorsionForce method)  getEnergyTermParameters() (simtk.openmm.openmm.CustomGBForce method)  getEpsh() (simtk.openmm.openmm.AmoebaWcaDispersionForce method)  getEpsilonCombiningRule() (simtk.openmm.openmm.AmoebaVdwForce method)  getEpso() (simtk.openmm.openmm.AmoebaWcaDispersionForce method)  getErrorTolerance() (simtk.openmm.openmm.VariableLangevinIntegrator method)  (simtk.openmm.openmm.VariableVerletIntegrator method)  getETotal() (simtk.openmm.amd.DualAMDIntegrator method)  getEwaldErrorTolerance() (simtk.openmm.openmm.AmoebaMultipoleForce method)  (simtk.openmm.openmm.NonbondedForce method)  getExceptionParameters() (simtk.openmm.openmm.NonbondedForce method)  getExclusionParticles() (simtk.openmm.openmm.CustomGBForce method)  (simtk.openmm.openmm.CustomHbondForce method)  (simtk.openmm.openmm.CustomManyParticleForce method)  (simtk.openmm.openmm.CustomNonbondedForce method)  getExtrapolationCoefficients() (simtk.openmm.openmm.AmoebaMultipoleForce method)  getForce() (simtk.openmm.openmm.System method)  getForceGroup() (simtk.openmm.openmm.AmoebaAngleForce method)  (simtk.openmm.openmm.AmoebaBondForce method)  (simtk.openmm.openmm.AmoebaGeneralizedKirkwoodForce method)  (simtk.openmm.openmm.AmoebaInPlaneAngleForce method)  (simtk.openmm.openmm.AmoebaMultipoleForce method)  (simtk.openmm.openmm.AmoebaOutOfPlaneBendForce method)  (simtk.openmm.openmm.AmoebaPiTorsionForce method)  (simtk.openmm.openmm.AmoebaStretchBendForce method)  (simtk.openmm.openmm.AmoebaTorsionTorsionForce method)  (simtk.openmm.openmm.AmoebaVdwForce method)  (simtk.openmm.openmm.AmoebaWcaDispersionForce method)  (simtk.openmm.openmm.AndersenThermostat method)  (simtk.openmm.openmm.CMAPTorsionForce method)  (simtk.openmm.openmm.CMMotionRemover method)  (simtk.openmm.openmm.CustomAngleForce method)  (simtk.openmm.openmm.CustomBondForce method)  (simtk.openmm.openmm.CustomCentroidBondForce method)  (simtk.openmm.openmm.CustomCompoundBondForce method)  (simtk.openmm.openmm.CustomExternalForce method)  (simtk.openmm.openmm.CustomGBForce method)  (simtk.openmm.openmm.CustomHbondForce method)  (simtk.openmm.openmm.CustomManyParticleForce method)  (simtk.openmm.openmm.CustomNonbondedForce method)  (simtk.openmm.openmm.CustomTorsionForce method)  (simtk.openmm.openmm.DrudeForce method)  (simtk.openmm.openmm.Force method)  (simtk.openmm.openmm.GBSAOBCForce method)  (simtk.openmm.openmm.HarmonicAngleForce method)  (simtk.openmm.openmm.HarmonicBondForce method)  (simtk.openmm.openmm.MonteCarloAnisotropicBarostat method)  (simtk.openmm.openmm.MonteCarloBarostat method)  (simtk.openmm.openmm.MonteCarloMembraneBarostat method)  (simtk.openmm.openmm.NonbondedForce method)  (simtk.openmm.openmm.PeriodicTorsionForce method)  (simtk.openmm.openmm.RBTorsionForce method)  (simtk.openmm.openmm.RPMDMonteCarloBarostat method)  getForces() (simtk.openmm.openmm.State method)  (simtk.openmm.openmm.System method)  getFrequency() (simtk.openmm.openmm.CMMotionRemover method)  (simtk.openmm.openmm.MonteCarloAnisotropicBarostat method)  (simtk.openmm.openmm.MonteCarloBarostat method)  (simtk.openmm.openmm.MonteCarloMembraneBarostat method)  (simtk.openmm.openmm.RPMDMonteCarloBarostat method)  getFriction() (simtk.openmm.openmm.BrownianIntegrator method)  (simtk.openmm.openmm.DrudeLangevinIntegrator method)  (simtk.openmm.openmm.LangevinIntegrator method)  (simtk.openmm.openmm.RPMDIntegrator method)  (simtk.openmm.openmm.VariableLangevinIntegrator method)  getFunctionParameters() (simtk.openmm.openmm.Continuous1DFunction method)  (simtk.openmm.openmm.Continuous2DFunction method)  (simtk.openmm.openmm.Continuous3DFunction method)  (simtk.openmm.openmm.CustomCompoundBondForce method)  (simtk.openmm.openmm.CustomGBForce method)  (simtk.openmm.openmm.CustomHbondForce method)  (simtk.openmm.openmm.CustomNonbondedForce method)  (simtk.openmm.openmm.Discrete1DFunction method)  (simtk.openmm.openmm.Discrete2DFunction method)  (simtk.openmm.openmm.Discrete3DFunction method)  getGenerators() (simtk.openmm.app.forcefield.ForceField method)  getGlobalParameterDefaultValue() (simtk.openmm.openmm.CustomAngleForce method)  (simtk.openmm.openmm.CustomBondForce method)  (simtk.openmm.openmm.CustomCentroidBondForce method)  (simtk.openmm.openmm.CustomCompoundBondForce method)  (simtk.openmm.openmm.CustomExternalForce method)  (simtk.openmm.openmm.CustomGBForce method)  (simtk.openmm.openmm.CustomHbondForce method)  (simtk.openmm.openmm.CustomManyParticleForce method)  (simtk.openmm.openmm.CustomNonbondedForce method)  (simtk.openmm.openmm.CustomTorsionForce method)  getGlobalParameterName() (simtk.openmm.openmm.CustomAngleForce method)  (simtk.openmm.openmm.CustomBondForce method)  (simtk.openmm.openmm.CustomCentroidBondForce method)  (simtk.openmm.openmm.CustomCompoundBondForce method)  (simtk.openmm.openmm.CustomExternalForce method)  (simtk.openmm.openmm.CustomGBForce method)  (simtk.openmm.openmm.CustomHbondForce method)  (simtk.openmm.openmm.CustomManyParticleForce method)  (simtk.openmm.openmm.CustomNonbondedForce method)  (simtk.openmm.openmm.CustomTorsionForce method)  getGlobalVariable() (simtk.openmm.amd.AMDForceGroupIntegrator method)  (simtk.openmm.amd.AMDIntegrator method)  (simtk.openmm.amd.DualAMDIntegrator method)  (simtk.openmm.mtsintegrator.MTSIntegrator method)  (simtk.openmm.openmm.CustomIntegrator method)  getGlobalVariableByName() (simtk.openmm.amd.AMDForceGroupIntegrator method)  (simtk.openmm.amd.AMDIntegrator method)  (simtk.openmm.amd.DualAMDIntegrator method)  (simtk.openmm.mtsintegrator.MTSIntegrator method)  (simtk.openmm.openmm.CustomIntegrator method)  getGlobalVariableName() (simtk.openmm.amd.AMDForceGroupIntegrator method)  (simtk.openmm.amd.AMDIntegrator method)  (simtk.openmm.amd.DualAMDIntegrator method)  (simtk.openmm.mtsintegrator.MTSIntegrator method)  (simtk.openmm.openmm.CustomIntegrator method)  getGroupParameters() (simtk.openmm.openmm.CustomCentroidBondForce method)  getIncludeCavityTerm() (simtk.openmm.openmm.AmoebaGeneralizedKirkwoodForce method)  getInducedDipoles() (simtk.openmm.openmm.AmoebaMultipoleForce method)  getIntegrator() (simtk.openmm.openmm.CompoundIntegrator method)  (simtk.openmm.openmm.Context method)  getInteractionGroupParameters() (simtk.openmm.openmm.CustomNonbondedForce method)  getIntProperty() (simtk.openmm.openmm.SerializationNode method)  getKineticEnergy() (simtk.openmm.openmm.State method)  getKineticEnergyExpression() (simtk.openmm.amd.AMDForceGroupIntegrator method)  (simtk.openmm.amd.AMDIntegrator method)  (simtk.openmm.amd.DualAMDIntegrator method)  (simtk.openmm.mtsintegrator.MTSIntegrator method)  (simtk.openmm.openmm.CustomIntegrator method)  getLocalPosition() (simtk.openmm.openmm.LocalCoordinatesSite method)  getMapParameters() (simtk.openmm.openmm.CMAPTorsionForce method)  getMatchingTemplates() (simtk.openmm.app.forcefield.ForceField method)  getMaxDrudeDistance() (simtk.openmm.openmm.DrudeLangevinIntegrator method)  getMinimizationErrorTolerance() (simtk.openmm.openmm.DrudeSCFIntegrator method)  getMolecules() (simtk.openmm.openmm.Context method)  getMultipoleParameters() (simtk.openmm.openmm.AmoebaMultipoleForce method)  getMutualInducedMaxIterations() (simtk.openmm.openmm.AmoebaMultipoleForce method)  getMutualInducedTargetEpsilon() (simtk.openmm.openmm.AmoebaMultipoleForce method)  getName() (simtk.openmm.openmm.Platform method)  (simtk.openmm.openmm.SerializationNode method)  getNonbondedMethod() (simtk.openmm.openmm.AmoebaMultipoleForce method)  (simtk.openmm.openmm.AmoebaVdwForce method)  (simtk.openmm.openmm.CustomGBForce method)  (simtk.openmm.openmm.CustomHbondForce method)  (simtk.openmm.openmm.CustomManyParticleForce method)  (simtk.openmm.openmm.CustomNonbondedForce method)  (simtk.openmm.openmm.GBSAOBCForce method)  (simtk.openmm.openmm.NonbondedForce method)  getNumAcceptors() (simtk.openmm.openmm.CustomHbondForce method)  getNumAngles() (simtk.openmm.openmm.AmoebaAngleForce method)  (simtk.openmm.openmm.AmoebaInPlaneAngleForce method)  (simtk.openmm.openmm.CustomAngleForce method)  (simtk.openmm.openmm.HarmonicAngleForce method)  getNumAtoms() (simtk.openmm.app.topology.Topology method)  getNumBonds() (simtk.openmm.openmm.AmoebaBondForce method)  (simtk.openmm.openmm.CustomBondForce method)  (simtk.openmm.openmm.CustomCentroidBondForce method)  (simtk.openmm.openmm.CustomCompoundBondForce method)  (simtk.openmm.openmm.HarmonicBondForce method)  getNumChains() (simtk.openmm.app.topology.Topology method)  getNumComputations() (simtk.openmm.amd.AMDForceGroupIntegrator method)  (simtk.openmm.amd.AMDIntegrator method)  (simtk.openmm.amd.DualAMDIntegrator method)  (simtk.openmm.mtsintegrator.MTSIntegrator method)  (simtk.openmm.openmm.CustomIntegrator method)  getNumComputedValues() (simtk.openmm.openmm.CustomGBForce method)  getNumConstraints() (simtk.openmm.openmm.System method)  getNumCopies() (simtk.openmm.openmm.RPMDIntegrator method)  getNumDonors() (simtk.openmm.openmm.CustomHbondForce method)  getNumEnergyTerms() (simtk.openmm.openmm.CustomGBForce method)  getNumExceptions() (simtk.openmm.openmm.NonbondedForce method)  getNumExclusions() (simtk.openmm.openmm.CustomGBForce method)  (simtk.openmm.openmm.CustomHbondForce method)  (simtk.openmm.openmm.CustomManyParticleForce method)  (simtk.openmm.openmm.CustomNonbondedForce method)  getNumForces() (simtk.openmm.openmm.System method)  getNumFrames() (simtk.openmm.app.gromacsgrofile.GromacsGroFile method)  (simtk.openmm.app.pdbfile.PDBFile method)  (simtk.openmm.app.pdbxfile.PDBxFile method)  getNumFunctions() (simtk.openmm.openmm.CustomCentroidBondForce method)  (simtk.openmm.openmm.CustomCompoundBondForce method)  (simtk.openmm.openmm.CustomGBForce method)  (simtk.openmm.openmm.CustomHbondForce method)  (simtk.openmm.openmm.CustomNonbondedForce method)  getNumGlobalParameters() (simtk.openmm.openmm.CustomAngleForce method)  (simtk.openmm.openmm.CustomBondForce method)  (simtk.openmm.openmm.CustomCentroidBondForce method)  (simtk.openmm.openmm.CustomCompoundBondForce method)  (simtk.openmm.openmm.CustomExternalForce method)  (simtk.openmm.openmm.CustomGBForce method)  (simtk.openmm.openmm.CustomHbondForce method)  (simtk.openmm.openmm.CustomManyParticleForce method)  (simtk.openmm.openmm.CustomNonbondedForce method)  (simtk.openmm.openmm.CustomTorsionForce method)  getNumGlobalVariables() (simtk.openmm.amd.AMDForceGroupIntegrator method)  (simtk.openmm.amd.AMDIntegrator method)  (simtk.openmm.amd.DualAMDIntegrator method)  (simtk.openmm.mtsintegrator.MTSIntegrator method)  (simtk.openmm.openmm.CustomIntegrator method)  getNumGroups() (simtk.openmm.openmm.CustomCentroidBondForce method) | getNumGroupsPerBond() (simtk.openmm.openmm.CustomCentroidBondForce method)  getNumIntegrators() (simtk.openmm.openmm.CompoundIntegrator method)  getNumInteractionGroups() (simtk.openmm.openmm.CustomNonbondedForce method)  getNumMaps() (simtk.openmm.openmm.CMAPTorsionForce method)  getNumMultipoles() (simtk.openmm.openmm.AmoebaMultipoleForce method)  getNumOutOfPlaneBends() (simtk.openmm.openmm.AmoebaOutOfPlaneBendForce method)  getNumParticles() (simtk.openmm.openmm.AmoebaGeneralizedKirkwoodForce method)  (simtk.openmm.openmm.AmoebaVdwForce method)  (simtk.openmm.openmm.AmoebaWcaDispersionForce method)  (simtk.openmm.openmm.CustomExternalForce method)  (simtk.openmm.openmm.CustomGBForce method)  (simtk.openmm.openmm.CustomManyParticleForce method)  (simtk.openmm.openmm.CustomNonbondedForce method)  (simtk.openmm.openmm.DrudeForce method)  (simtk.openmm.openmm.GBSAOBCForce method)  (simtk.openmm.openmm.LocalCoordinatesSite method)  (simtk.openmm.openmm.NonbondedForce method)  (simtk.openmm.openmm.OutOfPlaneSite method)  (simtk.openmm.openmm.System method)  (simtk.openmm.openmm.ThreeParticleAverageSite method)  (simtk.openmm.openmm.TwoParticleAverageSite method)  (simtk.openmm.openmm.VirtualSite method)  getNumParticlesPerBond() (simtk.openmm.openmm.CustomCompoundBondForce method)  getNumParticlesPerSet() (simtk.openmm.openmm.CustomManyParticleForce method)  getNumPerAcceptorParameters() (simtk.openmm.openmm.CustomHbondForce method)  getNumPerAngleParameters() (simtk.openmm.openmm.CustomAngleForce method)  getNumPerBondParameters() (simtk.openmm.openmm.CustomBondForce method)  (simtk.openmm.openmm.CustomCentroidBondForce method)  (simtk.openmm.openmm.CustomCompoundBondForce method)  getNumPerDofVariables() (simtk.openmm.amd.AMDForceGroupIntegrator method)  (simtk.openmm.amd.AMDIntegrator method)  (simtk.openmm.amd.DualAMDIntegrator method)  (simtk.openmm.mtsintegrator.MTSIntegrator method)  (simtk.openmm.openmm.CustomIntegrator method)  getNumPerDonorParameters() (simtk.openmm.openmm.CustomHbondForce method)  getNumPerParticleParameters() (simtk.openmm.openmm.CustomExternalForce method)  (simtk.openmm.openmm.CustomGBForce method)  (simtk.openmm.openmm.CustomManyParticleForce method)  (simtk.openmm.openmm.CustomNonbondedForce method)  getNumPerTorsionParameters() (simtk.openmm.openmm.CustomTorsionForce method)  getNumPiTorsions() (simtk.openmm.openmm.AmoebaPiTorsionForce method)  getNumPlatforms() (simtk.openmm.openmm.Platform static method)  getNumResidues() (simtk.openmm.app.topology.Topology method)  getNumScreenedPairs() (simtk.openmm.openmm.DrudeForce method)  getNumStretchBends() (simtk.openmm.openmm.AmoebaStretchBendForce method)  getNumTabulatedFunctions() (simtk.openmm.openmm.CustomCentroidBondForce method)  (simtk.openmm.openmm.CustomCompoundBondForce method)  (simtk.openmm.openmm.CustomGBForce method)  (simtk.openmm.openmm.CustomHbondForce method)  (simtk.openmm.openmm.CustomManyParticleForce method)  (simtk.openmm.openmm.CustomNonbondedForce method)  getNumTorsions() (simtk.openmm.openmm.CMAPTorsionForce method)  (simtk.openmm.openmm.CustomTorsionForce method)  (simtk.openmm.openmm.PeriodicTorsionForce method)  (simtk.openmm.openmm.RBTorsionForce method)  getNumTorsionTorsionGrids() (simtk.openmm.openmm.AmoebaTorsionTorsionForce method)  getNumTorsionTorsions() (simtk.openmm.openmm.AmoebaTorsionTorsionForce method)  getOpenMMVersion() (simtk.openmm.openmm.Platform static method)  getOriginWeights() (simtk.openmm.openmm.LocalCoordinatesSite method)  getOutOfPlaneBendParameters() (simtk.openmm.openmm.AmoebaOutOfPlaneBendForce method)  getParameter() (simtk.openmm.openmm.Context method)  getParameters() (simtk.openmm.openmm.Context method)  (simtk.openmm.openmm.State method)  getParticle() (simtk.openmm.openmm.LocalCoordinatesSite method)  (simtk.openmm.openmm.OutOfPlaneSite method)  (simtk.openmm.openmm.ThreeParticleAverageSite method)  (simtk.openmm.openmm.TwoParticleAverageSite method)  (simtk.openmm.openmm.VirtualSite method)  getParticleExclusions() (simtk.openmm.openmm.AmoebaVdwForce method)  getParticleMass() (simtk.openmm.openmm.System method)  getParticleParameters() (simtk.openmm.openmm.AmoebaGeneralizedKirkwoodForce method)  (simtk.openmm.openmm.AmoebaVdwForce method)  (simtk.openmm.openmm.AmoebaWcaDispersionForce method)  (simtk.openmm.openmm.CustomExternalForce method)  (simtk.openmm.openmm.CustomGBForce method)  (simtk.openmm.openmm.CustomManyParticleForce method)  (simtk.openmm.openmm.CustomNonbondedForce method)  (simtk.openmm.openmm.DrudeForce method)  (simtk.openmm.openmm.GBSAOBCForce method)  (simtk.openmm.openmm.NonbondedForce method)  getPerAcceptorParameterName() (simtk.openmm.openmm.CustomHbondForce method)  getPerAngleParameterName() (simtk.openmm.openmm.CustomAngleForce method)  getPerBondParameterName() (simtk.openmm.openmm.CustomBondForce method)  (simtk.openmm.openmm.CustomCentroidBondForce method)  (simtk.openmm.openmm.CustomCompoundBondForce method)  getPerDofVariable() (simtk.openmm.amd.AMDForceGroupIntegrator method)  (simtk.openmm.amd.AMDIntegrator method)  (simtk.openmm.amd.DualAMDIntegrator method)  (simtk.openmm.mtsintegrator.MTSIntegrator method)  (simtk.openmm.openmm.CustomIntegrator method)  getPerDofVariableByName() (simtk.openmm.amd.AMDForceGroupIntegrator method)  (simtk.openmm.amd.AMDIntegrator method)  (simtk.openmm.amd.DualAMDIntegrator method)  (simtk.openmm.mtsintegrator.MTSIntegrator method)  (simtk.openmm.openmm.CustomIntegrator method)  getPerDofVariableName() (simtk.openmm.amd.AMDForceGroupIntegrator method)  (simtk.openmm.amd.AMDIntegrator method)  (simtk.openmm.amd.DualAMDIntegrator method)  (simtk.openmm.mtsintegrator.MTSIntegrator method)  (simtk.openmm.openmm.CustomIntegrator method)  getPerDonorParameterName() (simtk.openmm.openmm.CustomHbondForce method)  getPeriodicBoxVectors() (simtk.openmm.app.gromacsgrofile.GromacsGroFile method)  (simtk.openmm.app.topology.Topology method)  (simtk.openmm.openmm.State method)  getPeriodicBoxVolume() (simtk.openmm.openmm.State method)  getPermutationMode() (simtk.openmm.openmm.CustomManyParticleForce method)  getPerParticleParameterName() (simtk.openmm.openmm.CustomExternalForce method)  (simtk.openmm.openmm.CustomGBForce method)  (simtk.openmm.openmm.CustomManyParticleForce method)  (simtk.openmm.openmm.CustomNonbondedForce method)  getPerTorsionParameterName() (simtk.openmm.openmm.CustomTorsionForce method)  getPiTorsionParameters() (simtk.openmm.openmm.AmoebaPiTorsionForce method)  getPlatform() (simtk.openmm.openmm.Context method)  (simtk.openmm.openmm.Platform static method)  getPlatformByName() (simtk.openmm.openmm.Platform static method)  getPluginLoadFailures() (simtk.openmm.openmm.Platform static method)  getPmeBSplineOrder() (simtk.openmm.openmm.AmoebaMultipoleForce method)  getPmeGridDimensions() (simtk.openmm.openmm.AmoebaMultipoleForce method)  getPMEParameters() (simtk.openmm.openmm.NonbondedForce method)  getPMEParametersInContext() (simtk.openmm.openmm.AmoebaMultipoleForce method)  (simtk.openmm.openmm.NonbondedForce method)  getPolarizationType() (simtk.openmm.openmm.AmoebaMultipoleForce method)  getPositions() (simtk.openmm.app.amberinpcrdfile.AmberInpcrdFile method)  (simtk.openmm.app.desmonddmsfile.DesmondDMSFile method)  (simtk.openmm.app.gromacsgrofile.GromacsGroFile method)  (simtk.openmm.app.modeller.Modeller method)  (simtk.openmm.app.pdbfile.PDBFile method)  (simtk.openmm.app.pdbxfile.PDBxFile method)  (simtk.openmm.openmm.State method)  getPotentialEnergy() (simtk.openmm.openmm.State method)  getProbeRadius() (simtk.openmm.openmm.AmoebaGeneralizedKirkwoodForce method)  getProperties() (simtk.openmm.openmm.SerializationNode method)  getPropertyDefaultValue() (simtk.openmm.openmm.Platform method)  getPropertyNames() (simtk.openmm.openmm.Platform method)  getPropertyValue() (simtk.openmm.openmm.Platform method)  getProvenance() (simtk.openmm.app.desmonddmsfile.DesmondDMSFile method)  getProxy() (simtk.openmm.openmm.SerializationProxy static method)  getRandomNumberSeed() (simtk.openmm.amd.AMDForceGroupIntegrator method)  (simtk.openmm.amd.AMDIntegrator method)  (simtk.openmm.amd.DualAMDIntegrator method)  (simtk.openmm.mtsintegrator.MTSIntegrator method)  (simtk.openmm.openmm.AndersenThermostat method)  (simtk.openmm.openmm.BrownianIntegrator method)  (simtk.openmm.openmm.CustomIntegrator method)  (simtk.openmm.openmm.DrudeLangevinIntegrator method)  (simtk.openmm.openmm.LangevinIntegrator method)  (simtk.openmm.openmm.MonteCarloAnisotropicBarostat method)  (simtk.openmm.openmm.MonteCarloBarostat method)  (simtk.openmm.openmm.MonteCarloMembraneBarostat method)  (simtk.openmm.openmm.RPMDIntegrator method)  (simtk.openmm.openmm.RPMDMonteCarloBarostat method)  (simtk.openmm.openmm.VariableLangevinIntegrator method)  getReactionFieldDielectric() (simtk.openmm.openmm.NonbondedForce method)  getReciprocalSpaceForceGroup() (simtk.openmm.openmm.NonbondedForce method)  getRminh() (simtk.openmm.openmm.AmoebaWcaDispersionForce method)  getRmino() (simtk.openmm.openmm.AmoebaWcaDispersionForce method)  getScaleX() (simtk.openmm.openmm.MonteCarloAnisotropicBarostat method)  getScaleY() (simtk.openmm.openmm.MonteCarloAnisotropicBarostat method)  getScaleZ() (simtk.openmm.openmm.MonteCarloAnisotropicBarostat method)  getScreenedPairParameters() (simtk.openmm.openmm.DrudeForce method)  getShctd() (simtk.openmm.openmm.AmoebaWcaDispersionForce method)  getSigmaCombiningRule() (simtk.openmm.openmm.AmoebaVdwForce method)  getSlevy() (simtk.openmm.openmm.AmoebaWcaDispersionForce method)  getSoluteDielectric() (simtk.openmm.openmm.AmoebaGeneralizedKirkwoodForce method)  (simtk.openmm.openmm.GBSAOBCForce method)  getSolventDielectric() (simtk.openmm.openmm.AmoebaGeneralizedKirkwoodForce method)  (simtk.openmm.openmm.GBSAOBCForce method)  getSpeed() (simtk.openmm.openmm.Platform method)  getState() (simtk.openmm.openmm.Context method)  (simtk.openmm.openmm.RPMDIntegrator method)  getStepSize() (simtk.openmm.amd.AMDForceGroupIntegrator method)  (simtk.openmm.amd.AMDIntegrator method)  (simtk.openmm.amd.DualAMDIntegrator method)  (simtk.openmm.mtsintegrator.MTSIntegrator method)  (simtk.openmm.openmm.BrownianIntegrator method)  (simtk.openmm.openmm.CompoundIntegrator method)  (simtk.openmm.openmm.CustomIntegrator method)  (simtk.openmm.openmm.DrudeLangevinIntegrator method)  (simtk.openmm.openmm.DrudeSCFIntegrator method)  (simtk.openmm.openmm.Integrator method)  (simtk.openmm.openmm.LangevinIntegrator method)  (simtk.openmm.openmm.RPMDIntegrator method)  (simtk.openmm.openmm.VariableLangevinIntegrator method)  (simtk.openmm.openmm.VariableVerletIntegrator method)  (simtk.openmm.openmm.VerletIntegrator method)  getStretchBendParameters() (simtk.openmm.openmm.AmoebaStretchBendForce method)  getStringProperty() (simtk.openmm.openmm.SerializationNode method)  getSurfaceAreaEnergy() (simtk.openmm.openmm.GBSAOBCForce method)  getSurfaceAreaFactor() (simtk.openmm.openmm.AmoebaGeneralizedKirkwoodForce method)  getSwitchingDistance() (simtk.openmm.openmm.CustomNonbondedForce method)  (simtk.openmm.openmm.NonbondedForce method)  getSystem() (simtk.openmm.openmm.Context method)  getSystemMultipoleMoments() (simtk.openmm.openmm.AmoebaMultipoleForce method)  getTabulatedFunction() (simtk.openmm.openmm.CustomCentroidBondForce method)  (simtk.openmm.openmm.CustomCompoundBondForce method)  (simtk.openmm.openmm.CustomGBForce method)  (simtk.openmm.openmm.CustomHbondForce method)  (simtk.openmm.openmm.CustomManyParticleForce method)  (simtk.openmm.openmm.CustomNonbondedForce method)  getTabulatedFunctionName() (simtk.openmm.openmm.CustomCentroidBondForce method)  (simtk.openmm.openmm.CustomCompoundBondForce method)  (simtk.openmm.openmm.CustomGBForce method)  (simtk.openmm.openmm.CustomHbondForce method)  (simtk.openmm.openmm.CustomManyParticleForce method)  (simtk.openmm.openmm.CustomNonbondedForce method)  getTemperature() (simtk.openmm.openmm.BrownianIntegrator method)  (simtk.openmm.openmm.DrudeLangevinIntegrator method)  (simtk.openmm.openmm.LangevinIntegrator method)  (simtk.openmm.openmm.MonteCarloAnisotropicBarostat method)  (simtk.openmm.openmm.MonteCarloBarostat method)  (simtk.openmm.openmm.MonteCarloMembraneBarostat method)  (simtk.openmm.openmm.RPMDIntegrator method)  (simtk.openmm.openmm.VariableLangevinIntegrator method)  getTime() (simtk.openmm.openmm.State method)  getTopology() (simtk.openmm.app.desmonddmsfile.DesmondDMSFile method)  (simtk.openmm.app.modeller.Modeller method)  (simtk.openmm.app.pdbfile.PDBFile method)  (simtk.openmm.app.pdbxfile.PDBxFile method)  getTorsionParameters() (simtk.openmm.openmm.CMAPTorsionForce method)  (simtk.openmm.openmm.CustomTorsionForce method)  (simtk.openmm.openmm.PeriodicTorsionForce method)  (simtk.openmm.openmm.RBTorsionForce method)  getTorsionTorsionGrid() (simtk.openmm.openmm.AmoebaTorsionTorsionForce method)  getTorsionTorsionParameters() (simtk.openmm.openmm.AmoebaTorsionTorsionForce method)  getTotalEnergy() (simtk.openmm.openmm.RPMDIntegrator method)  getTypeFilter() (simtk.openmm.openmm.CustomManyParticleForce method)  getTypeName() (simtk.openmm.openmm.SerializationProxy method)  getUnitCellDimensions() (simtk.openmm.app.gromacsgrofile.GromacsGroFile method)  (simtk.openmm.app.topology.Topology method)  getUnmatchedResidues() (simtk.openmm.app.forcefield.ForceField method)  getUseDispersionCorrection() (simtk.openmm.openmm.AmoebaVdwForce method)  (simtk.openmm.openmm.NonbondedForce method)  getUseLongRangeCorrection() (simtk.openmm.openmm.CustomNonbondedForce method)  getUseSwitchingFunction() (simtk.openmm.openmm.CustomNonbondedForce method)  (simtk.openmm.openmm.NonbondedForce method)  getVelocities() (simtk.openmm.app.amberinpcrdfile.AmberInpcrdFile method)  (simtk.openmm.openmm.State method)  getVirtualSite() (simtk.openmm.openmm.System method)  getWeight() (simtk.openmm.openmm.ThreeParticleAverageSite method)  (simtk.openmm.openmm.TwoParticleAverageSite method)  getWeight12() (simtk.openmm.openmm.OutOfPlaneSite method)  getWeight13() (simtk.openmm.openmm.OutOfPlaneSite method)  getWeightCross() (simtk.openmm.openmm.OutOfPlaneSite method)  getXWeights() (simtk.openmm.openmm.LocalCoordinatesSite method)  getXYMode() (simtk.openmm.openmm.MonteCarloMembraneBarostat method)  getYWeights() (simtk.openmm.openmm.LocalCoordinatesSite method)  getZMode() (simtk.openmm.openmm.MonteCarloMembraneBarostat method)  GromacsGroFile (class in simtk.openmm.app.gromacsgrofile)  GromacsTopFile (class in simtk.openmm.app.gromacstopfile) |

## H

|  |  |
| --- | --- |
| HarmonicAngleForce (class in simtk.openmm.openmm)  HarmonicBondForce (class in simtk.openmm.openmm) | hasProperty() (simtk.openmm.openmm.SerializationNode method)  helpful (simtk.openmm.app.charmmparameterset.CharmmParameterSet attribute) |

## I

|  |  |
| --- | --- |
| index() (simtk.openmm.vec3.Vec3 method)  Integrator (class in simtk.openmm.openmm) | internal\_bonds() (simtk.openmm.app.topology.Residue method)  isVirtualSite() (simtk.openmm.openmm.System method) |

## L

|  |  |
| --- | --- |
| LangevinIntegrator (class in simtk.openmm.openmm)  loadBondDefinitions() (simtk.openmm.app.topology.Topology static method)  loadCheckpoint() (simtk.openmm.app.simulation.Simulation method)  (simtk.openmm.openmm.Context method)  loadFile() (simtk.openmm.app.forcefield.ForceField method)  loadHydrogenDefinitions() (simtk.openmm.app.modeller.Modeller static method)  loadParameters() (simtk.openmm.app.charmmpsffile.CharmmPsfFile method) | loadPluginLibrary() (simtk.openmm.openmm.Platform static method)  loadPluginsFromDirectory() (simtk.openmm.openmm.Platform static method)  loadSet() (simtk.openmm.app.charmmparameterset.CharmmParameterSet class method)  loadState() (simtk.openmm.app.simulation.Simulation method)  LocalCoordinatesSite (class in simtk.openmm.openmm)  LocalEnergyMinimizer (class in simtk.openmm.openmm) |

## M

|  |  |
| --- | --- |
| minimize() (simtk.openmm.openmm.LocalEnergyMinimizer static method)  minimizeEnergy() (simtk.openmm.app.simulation.Simulation method)  Modeller (class in simtk.openmm.app.modeller)  MonteCarloAnisotropicBarostat (class in simtk.openmm.openmm) | MonteCarloBarostat (class in simtk.openmm.openmm)  MonteCarloMembraneBarostat (class in simtk.openmm.openmm)  MTSIntegrator (class in simtk.openmm.mtsintegrator) |

## N

|  |  |
| --- | --- |
| natom (simtk.openmm.app.charmmcrdfiles.CharmmCrdFile attribute)  (simtk.openmm.app.charmmcrdfiles.CharmmRstFile attribute) | NonbondedForce (class in simtk.openmm.openmm) |

## O

OutOfPlaneSite (class in simtk.openmm.openmm)

## P

|  |  |
| --- | --- |
| PDBFile (class in simtk.openmm.app.pdbfile)  PDBReporter (class in simtk.openmm.app.pdbreporter)  PDBxFile (class in simtk.openmm.app.pdbxfile)  PDBxReporter (class in simtk.openmm.app.pdbreporter)  PeriodicTorsionForce (class in simtk.openmm.openmm)  Platform (class in simtk.openmm.openmm) | positions (simtk.openmm.app.charmmcrdfiles.CharmmCrdFile attribute)  (simtk.openmm.app.charmmcrdfiles.CharmmRstFile attribute)  positionsold (simtk.openmm.app.charmmcrdfiles.CharmmRstFile attribute)  Pressure() (simtk.openmm.openmm.MonteCarloBarostat static method)  (simtk.openmm.openmm.MonteCarloMembraneBarostat static method)  (simtk.openmm.openmm.RPMDMonteCarloBarostat static method)  PressureX() (simtk.openmm.openmm.MonteCarloAnisotropicBarostat static method)  PressureY() (simtk.openmm.openmm.MonteCarloAnisotropicBarostat static method)  PressureZ() (simtk.openmm.openmm.MonteCarloAnisotropicBarostat static method) |

## R

|  |  |
| --- | --- |
| RBTorsionForce (class in simtk.openmm.openmm)  readParameterFile() (simtk.openmm.app.charmmparameterset.CharmmParameterSet method)  readStreamFile() (simtk.openmm.app.charmmparameterset.CharmmParameterSet method)  readTopologyFile() (simtk.openmm.app.charmmparameterset.CharmmParameterSet method)  registerAtomType() (simtk.openmm.app.forcefield.ForceField method)  registerGenerator() (simtk.openmm.app.forcefield.ForceField method)  registerPlatform() (simtk.openmm.openmm.Platform static method)  registerProxy() (simtk.openmm.openmm.SerializationProxy static method)  registerResidueTemplate() (simtk.openmm.app.forcefield.ForceField method)  registerScript() (simtk.openmm.app.forcefield.ForceField method)  registerTemplateGenerator() (simtk.openmm.app.forcefield.ForceField method) | reinitialize() (simtk.openmm.openmm.Context method)  removeConstraint() (simtk.openmm.openmm.System method)  removeForce() (simtk.openmm.openmm.System method)  report() (simtk.openmm.app.checkpointreporter.CheckpointReporter method)  (simtk.openmm.app.dcdreporter.DCDReporter method)  (simtk.openmm.app.pdbreporter.PDBReporter method)  (simtk.openmm.app.pdbreporter.PDBxReporter method)  (simtk.openmm.app.statedatareporter.StateDataReporter method)  Residue (class in simtk.openmm.app.topology)  residues() (simtk.openmm.app.topology.Chain method)  (simtk.openmm.app.topology.Topology method)  resname (simtk.openmm.app.charmmcrdfiles.CharmmCrdFile attribute)  (simtk.openmm.app.charmmcrdfiles.CharmmRstFile attribute)  RPMDIntegrator (class in simtk.openmm.openmm)  RPMDMonteCarloBarostat (class in simtk.openmm.openmm)  runForClockTime() (simtk.openmm.app.simulation.Simulation method) |

## S

|  |  |
| --- | --- |
| saveCheckpoint() (simtk.openmm.app.simulation.Simulation method)  saveState() (simtk.openmm.app.simulation.Simulation method)  SerializationNode (class in simtk.openmm.openmm)  SerializationProxy (class in simtk.openmm.openmm)  serialize() (simtk.openmm.openmm.SerializationProxy method)  (simtk.openmm.openmm.XmlSerializer static method)  serializeSystem() (simtk.openmm.openmm.XmlSerializer static method)  setAcceptorParameters() (simtk.openmm.openmm.CustomHbondForce method)  setAEwald() (simtk.openmm.openmm.AmoebaMultipoleForce method)  setAlpha() (simtk.openmm.amd.AMDIntegrator method)  setAlphaGroup() (simtk.openmm.amd.AMDForceGroupIntegrator method)  (simtk.openmm.amd.DualAMDIntegrator method)  setAlphaTotal() (simtk.openmm.amd.DualAMDIntegrator method)  setAmoebaGlobalAngleCubic() (simtk.openmm.openmm.AmoebaAngleForce method)  setAmoebaGlobalAnglePentic() (simtk.openmm.openmm.AmoebaAngleForce method)  setAmoebaGlobalAngleQuartic() (simtk.openmm.openmm.AmoebaAngleForce method)  setAmoebaGlobalAngleSextic() (simtk.openmm.openmm.AmoebaAngleForce method)  setAmoebaGlobalBondCubic() (simtk.openmm.openmm.AmoebaBondForce method)  setAmoebaGlobalBondQuartic() (simtk.openmm.openmm.AmoebaBondForce method)  setAmoebaGlobalInPlaneAngleCubic() (simtk.openmm.openmm.AmoebaInPlaneAngleForce method)  setAmoebaGlobalInPlaneAnglePentic() (simtk.openmm.openmm.AmoebaInPlaneAngleForce method)  setAmoebaGlobalInPlaneAngleQuartic() (simtk.openmm.openmm.AmoebaInPlaneAngleForce method)  setAmoebaGlobalInPlaneAngleSextic() (simtk.openmm.openmm.AmoebaInPlaneAngleForce method)  setAmoebaGlobalOutOfPlaneBendCubic() (simtk.openmm.openmm.AmoebaOutOfPlaneBendForce method)  setAmoebaGlobalOutOfPlaneBendPentic() (simtk.openmm.openmm.AmoebaOutOfPlaneBendForce method)  setAmoebaGlobalOutOfPlaneBendQuartic() (simtk.openmm.openmm.AmoebaOutOfPlaneBendForce method)  setAmoebaGlobalOutOfPlaneBendSextic() (simtk.openmm.openmm.AmoebaOutOfPlaneBendForce method)  setAngleParameters() (simtk.openmm.openmm.AmoebaAngleForce method)  (simtk.openmm.openmm.AmoebaInPlaneAngleForce method)  (simtk.openmm.openmm.CustomAngleForce method)  (simtk.openmm.openmm.HarmonicAngleForce method)  setApplyThermostat() (simtk.openmm.openmm.RPMDIntegrator method)  setAwater() (simtk.openmm.openmm.AmoebaWcaDispersionForce method)  setBondParameters() (simtk.openmm.openmm.AmoebaBondForce method)  (simtk.openmm.openmm.CustomBondForce method)  (simtk.openmm.openmm.CustomCentroidBondForce method)  (simtk.openmm.openmm.CustomCompoundBondForce method)  (simtk.openmm.openmm.HarmonicBondForce method)  setBoolProperty() (simtk.openmm.openmm.SerializationNode method)  setBox() (simtk.openmm.app.charmmpsffile.CharmmPsfFile method)  setComputedValueParameters() (simtk.openmm.openmm.CustomGBForce method)  setConstraintParameters() (simtk.openmm.openmm.System method)  setConstraintTolerance() (simtk.openmm.amd.AMDForceGroupIntegrator method)  (simtk.openmm.amd.AMDIntegrator method)  (simtk.openmm.amd.DualAMDIntegrator method)  (simtk.openmm.mtsintegrator.MTSIntegrator method)  (simtk.openmm.openmm.BrownianIntegrator method)  (simtk.openmm.openmm.CompoundIntegrator method)  (simtk.openmm.openmm.CustomIntegrator method)  (simtk.openmm.openmm.DrudeLangevinIntegrator method)  (simtk.openmm.openmm.DrudeSCFIntegrator method)  (simtk.openmm.openmm.Integrator method)  (simtk.openmm.openmm.LangevinIntegrator method)  (simtk.openmm.openmm.RPMDIntegrator method)  (simtk.openmm.openmm.VariableLangevinIntegrator method)  (simtk.openmm.openmm.VariableVerletIntegrator method)  (simtk.openmm.openmm.VerletIntegrator method)  setCovalentMap() (simtk.openmm.openmm.AmoebaMultipoleForce method)  setCurrentIntegrator() (simtk.openmm.openmm.CompoundIntegrator method)  setCutoff() (simtk.openmm.openmm.AmoebaVdwForce method)  setCutoffDistance() (simtk.openmm.openmm.AmoebaMultipoleForce method)  (simtk.openmm.openmm.CustomGBForce method)  (simtk.openmm.openmm.CustomHbondForce method)  (simtk.openmm.openmm.CustomManyParticleForce method)  (simtk.openmm.openmm.CustomNonbondedForce method)  (simtk.openmm.openmm.GBSAOBCForce method)  (simtk.openmm.openmm.NonbondedForce method)  setDefaultCollisionFrequency() (simtk.openmm.openmm.AndersenThermostat method)  setDefaultPeriodicBoxVectors() (simtk.openmm.openmm.System method)  setDefaultPressure() (simtk.openmm.openmm.MonteCarloAnisotropicBarostat method)  (simtk.openmm.openmm.MonteCarloBarostat method)  (simtk.openmm.openmm.MonteCarloMembraneBarostat method)  (simtk.openmm.openmm.RPMDMonteCarloBarostat method)  setDefaultSurfaceTension() (simtk.openmm.openmm.MonteCarloMembraneBarostat method)  setDefaultTemperature() (simtk.openmm.openmm.AndersenThermostat method)  setDispoff() (simtk.openmm.openmm.AmoebaWcaDispersionForce method)  setDonorParameters() (simtk.openmm.openmm.CustomHbondForce method)  setDoubleProperty() (simtk.openmm.openmm.SerializationNode method)  setDrudeFriction() (simtk.openmm.openmm.DrudeLangevinIntegrator method)  setDrudeTemperature() (simtk.openmm.openmm.DrudeLangevinIntegrator method)  setE() (simtk.openmm.amd.AMDIntegrator method)  setEGroup() (simtk.openmm.amd.AMDForceGroupIntegrator method)  (simtk.openmm.amd.DualAMDIntegrator method)  setEnergyFunction() (simtk.openmm.openmm.CustomAngleForce method)  (simtk.openmm.openmm.CustomBondForce method)  (simtk.openmm.openmm.CustomCentroidBondForce method)  (simtk.openmm.openmm.CustomCompoundBondForce method)  (simtk.openmm.openmm.CustomExternalForce method)  (simtk.openmm.openmm.CustomHbondForce method)  (simtk.openmm.openmm.CustomManyParticleForce method)  (simtk.openmm.openmm.CustomNonbondedForce method)  (simtk.openmm.openmm.CustomTorsionForce method)  setEnergyTermParameters() (simtk.openmm.openmm.CustomGBForce method)  setEpsh() (simtk.openmm.openmm.AmoebaWcaDispersionForce method)  setEpsilonCombiningRule() (simtk.openmm.openmm.AmoebaVdwForce method)  setEpso() (simtk.openmm.openmm.AmoebaWcaDispersionForce method)  setErrorTolerance() (simtk.openmm.openmm.VariableLangevinIntegrator method)  (simtk.openmm.openmm.VariableVerletIntegrator method)  setETotal() (simtk.openmm.amd.DualAMDIntegrator method)  setEwaldErrorTolerance() (simtk.openmm.openmm.AmoebaMultipoleForce method)  (simtk.openmm.openmm.NonbondedForce method)  setExceptionParameters() (simtk.openmm.openmm.NonbondedForce method)  setExclusionParticles() (simtk.openmm.openmm.CustomGBForce method)  (simtk.openmm.openmm.CustomHbondForce method)  (simtk.openmm.openmm.CustomManyParticleForce method)  (simtk.openmm.openmm.CustomNonbondedForce method)  setExtrapolationCoefficients() (simtk.openmm.openmm.AmoebaMultipoleForce method)  setForceGroup() (simtk.openmm.openmm.AmoebaAngleForce method)  (simtk.openmm.openmm.AmoebaBondForce method)  (simtk.openmm.openmm.AmoebaGeneralizedKirkwoodForce method)  (simtk.openmm.openmm.AmoebaInPlaneAngleForce method)  (simtk.openmm.openmm.AmoebaMultipoleForce method)  (simtk.openmm.openmm.AmoebaOutOfPlaneBendForce method)  (simtk.openmm.openmm.AmoebaPiTorsionForce method)  (simtk.openmm.openmm.AmoebaStretchBendForce method)  (simtk.openmm.openmm.AmoebaTorsionTorsionForce method)  (simtk.openmm.openmm.AmoebaVdwForce method)  (simtk.openmm.openmm.AmoebaWcaDispersionForce method)  (simtk.openmm.openmm.AndersenThermostat method)  (simtk.openmm.openmm.CMAPTorsionForce method)  (simtk.openmm.openmm.CMMotionRemover method)  (simtk.openmm.openmm.CustomAngleForce method)  (simtk.openmm.openmm.CustomBondForce method)  (simtk.openmm.openmm.CustomCentroidBondForce method)  (simtk.openmm.openmm.CustomCompoundBondForce method)  (simtk.openmm.openmm.CustomExternalForce method)  (simtk.openmm.openmm.CustomGBForce method)  (simtk.openmm.openmm.CustomHbondForce method)  (simtk.openmm.openmm.CustomManyParticleForce method)  (simtk.openmm.openmm.CustomNonbondedForce method)  (simtk.openmm.openmm.CustomTorsionForce method)  (simtk.openmm.openmm.DrudeForce method)  (simtk.openmm.openmm.Force method)  (simtk.openmm.openmm.GBSAOBCForce method)  (simtk.openmm.openmm.HarmonicAngleForce method)  (simtk.openmm.openmm.HarmonicBondForce method)  (simtk.openmm.openmm.MonteCarloAnisotropicBarostat method)  (simtk.openmm.openmm.MonteCarloBarostat method)  (simtk.openmm.openmm.MonteCarloMembraneBarostat method)  (simtk.openmm.openmm.NonbondedForce method)  (simtk.openmm.openmm.PeriodicTorsionForce method)  (simtk.openmm.openmm.RBTorsionForce method)  (simtk.openmm.openmm.RPMDMonteCarloBarostat method)  setFrequency() (simtk.openmm.openmm.CMMotionRemover method)  (simtk.openmm.openmm.MonteCarloAnisotropicBarostat method)  (simtk.openmm.openmm.MonteCarloBarostat method)  (simtk.openmm.openmm.MonteCarloMembraneBarostat method)  (simtk.openmm.openmm.RPMDMonteCarloBarostat method)  setFriction() (simtk.openmm.openmm.BrownianIntegrator method)  (simtk.openmm.openmm.DrudeLangevinIntegrator method)  (simtk.openmm.openmm.LangevinIntegrator method)  (simtk.openmm.openmm.RPMDIntegrator method)  (simtk.openmm.openmm.VariableLangevinIntegrator method)  setFunctionParameters() (simtk.openmm.openmm.Continuous1DFunction method)  (simtk.openmm.openmm.Continuous2DFunction method)  (simtk.openmm.openmm.Continuous3DFunction method)  (simtk.openmm.openmm.CustomCompoundBondForce method)  (simtk.openmm.openmm.CustomGBForce method)  (simtk.openmm.openmm.CustomHbondForce method)  (simtk.openmm.openmm.CustomNonbondedForce method)  (simtk.openmm.openmm.Discrete1DFunction method)  (simtk.openmm.openmm.Discrete2DFunction method)  (simtk.openmm.openmm.Discrete3DFunction method)  setGlobalParameterDefaultValue() (simtk.openmm.openmm.CustomAngleForce method)  (simtk.openmm.openmm.CustomBondForce method)  (simtk.openmm.openmm.CustomCentroidBondForce method)  (simtk.openmm.openmm.CustomCompoundBondForce method)  (simtk.openmm.openmm.CustomExternalForce method)  (simtk.openmm.openmm.CustomGBForce method)  (simtk.openmm.openmm.CustomHbondForce method)  (simtk.openmm.openmm.CustomManyParticleForce method)  (simtk.openmm.openmm.CustomNonbondedForce method)  (simtk.openmm.openmm.CustomTorsionForce method)  setGlobalParameterName() (simtk.openmm.openmm.CustomAngleForce method)  (simtk.openmm.openmm.CustomBondForce method)  (simtk.openmm.openmm.CustomCentroidBondForce method)  (simtk.openmm.openmm.CustomCompoundBondForce method)  (simtk.openmm.openmm.CustomExternalForce method)  (simtk.openmm.openmm.CustomGBForce method)  (simtk.openmm.openmm.CustomHbondForce method)  (simtk.openmm.openmm.CustomManyParticleForce method)  (simtk.openmm.openmm.CustomNonbondedForce method)  (simtk.openmm.openmm.CustomTorsionForce method)  setGlobalVariable() (simtk.openmm.amd.AMDForceGroupIntegrator method)  (simtk.openmm.amd.AMDIntegrator method)  (simtk.openmm.amd.DualAMDIntegrator method)  (simtk.openmm.mtsintegrator.MTSIntegrator method)  (simtk.openmm.openmm.CustomIntegrator method)  setGlobalVariableByName() (simtk.openmm.amd.AMDForceGroupIntegrator method)  (simtk.openmm.amd.AMDIntegrator method)  (simtk.openmm.amd.DualAMDIntegrator method)  (simtk.openmm.mtsintegrator.MTSIntegrator method)  (simtk.openmm.openmm.CustomIntegrator method)  setGroupParameters() (simtk.openmm.openmm.CustomCentroidBondForce method)  setIncludeCavityTerm() (simtk.openmm.openmm.AmoebaGeneralizedKirkwoodForce method)  setInteractionGroupParameters() (simtk.openmm.openmm.CustomNonbondedForce method)  setIntProperty() (simtk.openmm.openmm.SerializationNode method)  setKineticEnergyExpression() (simtk.openmm.amd.AMDForceGroupIntegrator method)  (simtk.openmm.amd.AMDIntegrator method)  (simtk.openmm.amd.DualAMDIntegrator method)  (simtk.openmm.mtsintegrator.MTSIntegrator method)  (simtk.openmm.openmm.CustomIntegrator method) | setMapParameters() (simtk.openmm.openmm.CMAPTorsionForce method)  setMaxDrudeDistance() (simtk.openmm.openmm.DrudeLangevinIntegrator method)  setMinimizationErrorTolerance() (simtk.openmm.openmm.DrudeSCFIntegrator method)  setMultipoleParameters() (simtk.openmm.openmm.AmoebaMultipoleForce method)  setMutualInducedMaxIterations() (simtk.openmm.openmm.AmoebaMultipoleForce method)  setMutualInducedTargetEpsilon() (simtk.openmm.openmm.AmoebaMultipoleForce method)  setName() (simtk.openmm.openmm.SerializationNode method)  setNonbondedMethod() (simtk.openmm.openmm.AmoebaMultipoleForce method)  (simtk.openmm.openmm.AmoebaVdwForce method)  (simtk.openmm.openmm.CustomGBForce method)  (simtk.openmm.openmm.CustomHbondForce method)  (simtk.openmm.openmm.CustomManyParticleForce method)  (simtk.openmm.openmm.CustomNonbondedForce method)  (simtk.openmm.openmm.GBSAOBCForce method)  (simtk.openmm.openmm.NonbondedForce method)  setOutOfPlaneBendParameters() (simtk.openmm.openmm.AmoebaOutOfPlaneBendForce method)  setParameter() (simtk.openmm.openmm.Context method)  setParticleExclusions() (simtk.openmm.openmm.AmoebaVdwForce method)  setParticleMass() (simtk.openmm.openmm.System method)  setParticleParameters() (simtk.openmm.openmm.AmoebaGeneralizedKirkwoodForce method)  (simtk.openmm.openmm.AmoebaVdwForce method)  (simtk.openmm.openmm.AmoebaWcaDispersionForce method)  (simtk.openmm.openmm.CustomExternalForce method)  (simtk.openmm.openmm.CustomGBForce method)  (simtk.openmm.openmm.CustomManyParticleForce method)  (simtk.openmm.openmm.CustomNonbondedForce method)  (simtk.openmm.openmm.DrudeForce method)  (simtk.openmm.openmm.GBSAOBCForce method)  (simtk.openmm.openmm.NonbondedForce method)  setPerAcceptorParameterName() (simtk.openmm.openmm.CustomHbondForce method)  setPerAngleParameterName() (simtk.openmm.openmm.CustomAngleForce method)  setPerBondParameterName() (simtk.openmm.openmm.CustomBondForce method)  (simtk.openmm.openmm.CustomCentroidBondForce method)  (simtk.openmm.openmm.CustomCompoundBondForce method)  setPerDofVariable() (simtk.openmm.amd.AMDForceGroupIntegrator method)  (simtk.openmm.amd.AMDIntegrator method)  (simtk.openmm.amd.DualAMDIntegrator method)  (simtk.openmm.mtsintegrator.MTSIntegrator method)  (simtk.openmm.openmm.CustomIntegrator method)  setPerDofVariableByName() (simtk.openmm.amd.AMDForceGroupIntegrator method)  (simtk.openmm.amd.AMDIntegrator method)  (simtk.openmm.amd.DualAMDIntegrator method)  (simtk.openmm.mtsintegrator.MTSIntegrator method)  (simtk.openmm.openmm.CustomIntegrator method)  setPerDonorParameterName() (simtk.openmm.openmm.CustomHbondForce method)  setPeriodicBoxVectors() (simtk.openmm.app.topology.Topology method)  (simtk.openmm.openmm.Context method)  setPermutationMode() (simtk.openmm.openmm.CustomManyParticleForce method)  setPerParticleParameterName() (simtk.openmm.openmm.CustomExternalForce method)  (simtk.openmm.openmm.CustomGBForce method)  (simtk.openmm.openmm.CustomManyParticleForce method)  (simtk.openmm.openmm.CustomNonbondedForce method)  setPerTorsionParameterName() (simtk.openmm.openmm.CustomTorsionForce method)  setPiTorsionParameters() (simtk.openmm.openmm.AmoebaPiTorsionForce method)  setPmeGridDimensions() (simtk.openmm.openmm.AmoebaMultipoleForce method)  setPMEParameters() (simtk.openmm.openmm.NonbondedForce method)  setPolarizationType() (simtk.openmm.openmm.AmoebaMultipoleForce method)  setPositions() (simtk.openmm.openmm.Context method)  (simtk.openmm.openmm.RPMDIntegrator method)  setProbeRadius() (simtk.openmm.openmm.AmoebaGeneralizedKirkwoodForce method)  setPropertyDefaultValue() (simtk.openmm.openmm.Platform method)  setPropertyValue() (simtk.openmm.openmm.Platform method)  setRandomNumberSeed() (simtk.openmm.amd.AMDForceGroupIntegrator method)  (simtk.openmm.amd.AMDIntegrator method)  (simtk.openmm.amd.DualAMDIntegrator method)  (simtk.openmm.mtsintegrator.MTSIntegrator method)  (simtk.openmm.openmm.AndersenThermostat method)  (simtk.openmm.openmm.BrownianIntegrator method)  (simtk.openmm.openmm.CustomIntegrator method)  (simtk.openmm.openmm.DrudeLangevinIntegrator method)  (simtk.openmm.openmm.LangevinIntegrator method)  (simtk.openmm.openmm.MonteCarloAnisotropicBarostat method)  (simtk.openmm.openmm.MonteCarloBarostat method)  (simtk.openmm.openmm.MonteCarloMembraneBarostat method)  (simtk.openmm.openmm.RPMDIntegrator method)  (simtk.openmm.openmm.RPMDMonteCarloBarostat method)  (simtk.openmm.openmm.VariableLangevinIntegrator method)  setReactionFieldDielectric() (simtk.openmm.openmm.NonbondedForce method)  setReciprocalSpaceForceGroup() (simtk.openmm.openmm.NonbondedForce method)  setRminh() (simtk.openmm.openmm.AmoebaWcaDispersionForce method)  setRmino() (simtk.openmm.openmm.AmoebaWcaDispersionForce method)  setScreenedPairParameters() (simtk.openmm.openmm.DrudeForce method)  setShctd() (simtk.openmm.openmm.AmoebaWcaDispersionForce method)  setSigmaCombiningRule() (simtk.openmm.openmm.AmoebaVdwForce method)  setSlevy() (simtk.openmm.openmm.AmoebaWcaDispersionForce method)  setSoluteDielectric() (simtk.openmm.openmm.AmoebaGeneralizedKirkwoodForce method)  (simtk.openmm.openmm.GBSAOBCForce method)  setSolventDielectric() (simtk.openmm.openmm.AmoebaGeneralizedKirkwoodForce method)  (simtk.openmm.openmm.GBSAOBCForce method)  setState() (simtk.openmm.openmm.Context method)  setStepSize() (simtk.openmm.amd.AMDForceGroupIntegrator method)  (simtk.openmm.amd.AMDIntegrator method)  (simtk.openmm.amd.DualAMDIntegrator method)  (simtk.openmm.mtsintegrator.MTSIntegrator method)  (simtk.openmm.openmm.BrownianIntegrator method)  (simtk.openmm.openmm.CompoundIntegrator method)  (simtk.openmm.openmm.CustomIntegrator method)  (simtk.openmm.openmm.DrudeLangevinIntegrator method)  (simtk.openmm.openmm.DrudeSCFIntegrator method)  (simtk.openmm.openmm.Integrator method)  (simtk.openmm.openmm.LangevinIntegrator method)  (simtk.openmm.openmm.RPMDIntegrator method)  (simtk.openmm.openmm.VariableLangevinIntegrator method)  (simtk.openmm.openmm.VariableVerletIntegrator method)  (simtk.openmm.openmm.VerletIntegrator method)  setStretchBendParameters() (simtk.openmm.openmm.AmoebaStretchBendForce method)  setStringProperty() (simtk.openmm.openmm.SerializationNode method)  setSurfaceAreaEnergy() (simtk.openmm.openmm.GBSAOBCForce method)  setSurfaceAreaFactor() (simtk.openmm.openmm.AmoebaGeneralizedKirkwoodForce method)  setSwitchingDistance() (simtk.openmm.openmm.CustomNonbondedForce method)  (simtk.openmm.openmm.NonbondedForce method)  setTemperature() (simtk.openmm.openmm.BrownianIntegrator method)  (simtk.openmm.openmm.DrudeLangevinIntegrator method)  (simtk.openmm.openmm.LangevinIntegrator method)  (simtk.openmm.openmm.MonteCarloAnisotropicBarostat method)  (simtk.openmm.openmm.MonteCarloBarostat method)  (simtk.openmm.openmm.MonteCarloMembraneBarostat method)  (simtk.openmm.openmm.RPMDIntegrator method)  (simtk.openmm.openmm.VariableLangevinIntegrator method)  setTime() (simtk.openmm.openmm.Context method)  setTorsionParameters() (simtk.openmm.openmm.CMAPTorsionForce method)  (simtk.openmm.openmm.CustomTorsionForce method)  (simtk.openmm.openmm.PeriodicTorsionForce method)  (simtk.openmm.openmm.RBTorsionForce method)  setTorsionTorsionGrid() (simtk.openmm.openmm.AmoebaTorsionTorsionForce method)  setTorsionTorsionParameters() (simtk.openmm.openmm.AmoebaTorsionTorsionForce method)  setTypeFilter() (simtk.openmm.openmm.CustomManyParticleForce method)  setUnitCellDimensions() (simtk.openmm.app.topology.Topology method)  setUseDispersionCorrection() (simtk.openmm.openmm.AmoebaVdwForce method)  (simtk.openmm.openmm.NonbondedForce method)  setUseLongRangeCorrection() (simtk.openmm.openmm.CustomNonbondedForce method)  setUseSwitchingFunction() (simtk.openmm.openmm.CustomNonbondedForce method)  (simtk.openmm.openmm.NonbondedForce method)  setVelocities() (simtk.openmm.openmm.Context method)  (simtk.openmm.openmm.RPMDIntegrator method)  setVelocitiesToTemperature() (simtk.openmm.openmm.Context method)  setVirtualSite() (simtk.openmm.openmm.System method)  setXYMode() (simtk.openmm.openmm.MonteCarloMembraneBarostat method)  setZMode() (simtk.openmm.openmm.MonteCarloMembraneBarostat method)  Simulation (class in simtk.openmm.app.simulation)  State (class in simtk.openmm.openmm)  StateDataReporter (class in simtk.openmm.app.statedatareporter)  step() (simtk.openmm.amd.AMDForceGroupIntegrator method)  (simtk.openmm.amd.AMDIntegrator method)  (simtk.openmm.amd.DualAMDIntegrator method)  (simtk.openmm.app.simulation.Simulation method)  (simtk.openmm.mtsintegrator.MTSIntegrator method)  (simtk.openmm.openmm.BrownianIntegrator method)  (simtk.openmm.openmm.CompoundIntegrator method)  (simtk.openmm.openmm.CustomIntegrator method)  (simtk.openmm.openmm.DrudeLangevinIntegrator method)  (simtk.openmm.openmm.DrudeSCFIntegrator method)  (simtk.openmm.openmm.Integrator method)  (simtk.openmm.openmm.LangevinIntegrator method)  (simtk.openmm.openmm.RPMDIntegrator method)  (simtk.openmm.openmm.VariableLangevinIntegrator method)  (simtk.openmm.openmm.VariableVerletIntegrator method)  (simtk.openmm.openmm.VerletIntegrator method)  stepTo() (simtk.openmm.openmm.VariableLangevinIntegrator method)  (simtk.openmm.openmm.VariableVerletIntegrator method)  supportsDoublePrecision() (simtk.openmm.openmm.Platform method)  supportsKernels() (simtk.openmm.openmm.Platform method)  SurfaceTension() (simtk.openmm.openmm.MonteCarloMembraneBarostat static method)  System (class in simtk.openmm.openmm)  system (simtk.openmm.app.charmmpsffile.CharmmPsfFile attribute) |

## T

|  |  |
| --- | --- |
| TabulatedFunction (class in simtk.openmm.openmm)  Temperature() (simtk.openmm.openmm.AndersenThermostat static method)  ThreeParticleAverageSite (class in simtk.openmm.openmm) | Topology (class in simtk.openmm.app.topology)  topology (simtk.openmm.app.charmmpsffile.CharmmPsfFile attribute)  TwoParticleAverageSite (class in simtk.openmm.openmm) |

## U

|  |  |
| --- | --- |
| updateParametersInContext() (simtk.openmm.openmm.AmoebaAngleForce method)  (simtk.openmm.openmm.AmoebaBondForce method)  (simtk.openmm.openmm.AmoebaGeneralizedKirkwoodForce method)  (simtk.openmm.openmm.AmoebaInPlaneAngleForce method)  (simtk.openmm.openmm.AmoebaMultipoleForce method)  (simtk.openmm.openmm.AmoebaOutOfPlaneBendForce method)  (simtk.openmm.openmm.AmoebaPiTorsionForce method)  (simtk.openmm.openmm.AmoebaStretchBendForce method)  (simtk.openmm.openmm.AmoebaVdwForce method)  (simtk.openmm.openmm.AmoebaWcaDispersionForce method)  (simtk.openmm.openmm.CMAPTorsionForce method)  (simtk.openmm.openmm.CustomAngleForce method)  (simtk.openmm.openmm.CustomBondForce method)  (simtk.openmm.openmm.CustomCentroidBondForce method)  (simtk.openmm.openmm.CustomCompoundBondForce method)  (simtk.openmm.openmm.CustomExternalForce method)  (simtk.openmm.openmm.CustomGBForce method)  (simtk.openmm.openmm.CustomHbondForce method)  (simtk.openmm.openmm.CustomManyParticleForce method)  (simtk.openmm.openmm.CustomNonbondedForce method)  (simtk.openmm.openmm.CustomTorsionForce method)  (simtk.openmm.openmm.DrudeForce method)  (simtk.openmm.openmm.GBSAOBCForce method)  (simtk.openmm.openmm.HarmonicAngleForce method)  (simtk.openmm.openmm.HarmonicBondForce method)  (simtk.openmm.openmm.NonbondedForce method)  (simtk.openmm.openmm.PeriodicTorsionForce method)  (simtk.openmm.openmm.RBTorsionForce method) | usesPeriodicBoundaryConditions() (simtk.openmm.openmm.AmoebaAngleForce method)  (simtk.openmm.openmm.AmoebaBondForce method)  (simtk.openmm.openmm.AmoebaGeneralizedKirkwoodForce method)  (simtk.openmm.openmm.AmoebaInPlaneAngleForce method)  (simtk.openmm.openmm.AmoebaMultipoleForce method)  (simtk.openmm.openmm.AmoebaOutOfPlaneBendForce method)  (simtk.openmm.openmm.AmoebaPiTorsionForce method)  (simtk.openmm.openmm.AmoebaStretchBendForce method)  (simtk.openmm.openmm.AmoebaTorsionTorsionForce method)  (simtk.openmm.openmm.AmoebaVdwForce method)  (simtk.openmm.openmm.AmoebaWcaDispersionForce method)  (simtk.openmm.openmm.AndersenThermostat method)  (simtk.openmm.openmm.CMAPTorsionForce method)  (simtk.openmm.openmm.CMMotionRemover method)  (simtk.openmm.openmm.CustomAngleForce method)  (simtk.openmm.openmm.CustomBondForce method)  (simtk.openmm.openmm.CustomCentroidBondForce method)  (simtk.openmm.openmm.CustomCompoundBondForce method)  (simtk.openmm.openmm.CustomExternalForce method)  (simtk.openmm.openmm.CustomGBForce method)  (simtk.openmm.openmm.CustomHbondForce method)  (simtk.openmm.openmm.CustomManyParticleForce method)  (simtk.openmm.openmm.CustomNonbondedForce method)  (simtk.openmm.openmm.CustomTorsionForce method)  (simtk.openmm.openmm.DrudeForce method)  (simtk.openmm.openmm.Force method)  (simtk.openmm.openmm.GBSAOBCForce method)  (simtk.openmm.openmm.HarmonicAngleForce method)  (simtk.openmm.openmm.HarmonicBondForce method)  (simtk.openmm.openmm.MonteCarloAnisotropicBarostat method)  (simtk.openmm.openmm.MonteCarloBarostat method)  (simtk.openmm.openmm.MonteCarloMembraneBarostat method)  (simtk.openmm.openmm.NonbondedForce method)  (simtk.openmm.openmm.PeriodicTorsionForce method)  (simtk.openmm.openmm.RBTorsionForce method)  (simtk.openmm.openmm.RPMDMonteCarloBarostat method)  (simtk.openmm.openmm.System method) |

## V

|  |  |
| --- | --- |
| VariableLangevinIntegrator (class in simtk.openmm.openmm)  VariableVerletIntegrator (class in simtk.openmm.openmm)  Vec3 (class in simtk.openmm.vec3) | velocities (simtk.openmm.app.charmmcrdfiles.CharmmRstFile attribute)  VerletIntegrator (class in simtk.openmm.openmm)  VirtualSite (class in simtk.openmm.openmm) |

## W

|  |  |
| --- | --- |
| writeFile() (simtk.openmm.app.pdbfile.PDBFile static method)  (simtk.openmm.app.pdbxfile.PDBxFile static method)  writeFooter() (simtk.openmm.app.pdbfile.PDBFile static method) | writeHeader() (simtk.openmm.app.pdbfile.PDBFile static method)  (simtk.openmm.app.pdbxfile.PDBxFile static method)  writeModel() (simtk.openmm.app.dcdfile.DCDFile method)  (simtk.openmm.app.pdbfile.PDBFile static method)  (simtk.openmm.app.pdbxfile.PDBxFile static method) |

## X

XmlSerializer (class in simtk.openmm.openmm)

High performance molecular simulation on GPUs


### Navigation

- Application Layer
- Library Layer

©2015, Stanford University and the Authors.
|
Powered by Sphinx 1.3.1
& Alabaster 0.7.7
